# AI-powered IC50 prediction for p53 inhibitors drug-target interaction via hybrid graph neural networks

**DOI:** 10.1007/s10822-025-00723-z

**Published:** 2025-12-24

**Authors:** Walaa H. El-Masry, Samar Monem, Nagy Ramadan Darwish, Aboul Ella Hassanein

**Affiliations:** 1 Information Systems and Technology Department, Faculty of Graduate Studies for Statistical Research, Cairo University, Giza, Egypt; 2 Mathematics and Computer Science Department, Faculty of Science, Beni-Suef University, Beni-Suef, Egypt; 3 Information Technology Department, Faculty of Computers and Artificial Intelligence, Cairo University, Giza, Egypt; 4 Scientific Research School of Egypt (SRSEG), Giza, Egypt

**Keywords:** Drug-target interaction, p53 Inhibitors, IC50, GCN, GAT, Deep learning, AI, Sustainable development, Hybrid model

## Abstract

In recent decades, the rapid pace of digital transformation marks a transformative era for the healthcare and pharmaceutical industries. The incorporation of innovative technology, specifically Artificial Intelligence (AI) and its derivatives, has driven significant innovation and greatly enhanced the efficiency of biomedical research and drug discovery processes. Among critical biological targets, the p53 protein is essential for controlling cell cycle regulation and tumor suppression. Although p53 has long been considered undruggable, recent research has revived interest in targeting it with novel therapeutics. In this paper, A novel Hybrid Drug-Target Interaction IC50 (HDTI-IC50) prediction model is proposes to predict IC50 values. The model integrates Graph Convolutional Networks (GCNs) as well as Graph Attention Networks (GATs) by sequentially stacking their hidden layers. This hybrid architecture leverages the strengths of both models. Specifically, GCNs are first applied to effectively capture local structural information and perform well under homophily assumptions. Then, GAT is learned to model long-range dependencies and handle heterophilic graphs. By integrating both, the model learns richer node representations and can adapt to diverse graph structures. Following these layers, a global pooling mechanism follows, which combines Global Max Pooling (GMP) and Global Average Pooling (GAP). Compared to related approaches, which mainly perform general IC50 prediction or binary activity classification, the proposed HDTI-IC50 model provides a unified framework specifically tailored for p53 inhibitors. Unlike previous approaches that rely on conventional molecular descriptors and overlook structural topology, our model utilizes graph-based representations to capture both local and global molecular relationships. By sequentially integrating GCN and GAT layers, the model effectively combines localized structural learning with attention-based feature refinement, resulting in improved representation capability and predictive performance. The dataset applied in this paper is obtained from the database of the Genomics of Drug Sensitivity in Cancer (GDSC). Model performance is evaluated using standard regression metrics, involving Mean Absolute Error (MAE), Root Mean Square Error (RMSE), and coefficient of determination (R^2^). The performance rate of MAE is 0.1, RMSE is 0.19, and R^2^ is 0.8 demonstrating superior performance compared to state-of-the-art methods. It also achieves an average inference time of 7.70 s. This paper proposes a HDTI-IC50 model to predict IC50 for p53inhibitors. Results from experiments indicate that the proposed HDTI-IC50 model outperforms individual GCN, GAT-based, and other related drug-target models as well as baseline regression models. demonstrating both its predictive accuracy and computational economy.

## Introduction

In the last few years, the number of medications authorized by the Food and Drug Administration (FDA) has diminished, despite significant advancements in biotechnology. To get medicine to market, a significant expenditure is required, ranging between $2 billion and $3 billion [1]. Furthermore, it typically requires a prolonged period roughly twelve years or more when a new medication is being developed and approved for release onto the market. The standard drug development process consists of verifying targets, identifying the proper molecule that interacts towards the target, assessing the product for efficiency as well as safety, and, in case of efficiency, moving through a drug endorsement process to finally reach the market [8]. Whenever hundreds of chemical substances are evaluated, the entire procedure can become costly and time-consuming, particularly when acquiring or synthesizing large chemical resources. Many of them ultimately fail to become therapeutic candidates or fail in clinical trials. To address these challenges, cheminformatics has been applied to various steps in drug discovery. Cheminformatics, also known as chemoinformatics or chemical informatics. It merges chemistry, computer science, as well as informatics to handle data on molecular constituents using computational analysis. Cheminformatics facilitates the storage, querying, and retrieval of large-scale chemical data, enabling the investigation of the connections among molecular structures, characteristics, as well as behaviors of molecules.

Investigating interactions between compounds and targets through biomedical research demands substantial resources such as financial investment, time, and human resource costs; the total amount of confirmed Drug Target Interactions (DTIs) is quite low. To conserve both time as well as resources to satisfy the demands of the current genesis, machine learning was developed and conducted towards the forecasting of interactions between drugs and their targets [39].

A drug is defined as a chemical compound that alters physiological or psychological functions in the human body and is essential for the diagnosis, treatment, and prevention of diseases. These compounds exert their effects by interacting with specific molecules in the body, referring to drug targets, which are most often proteins responsible for initiating and regulating cellular responses. Predicting drug-target interactions is an important area of research in drug discovery, repositioning, and understanding side effects or resistance. This process involves identifying connections between chemical substances and protein targets in the human body. However, conducting laboratory experiments to uncover these interactions is often time-consuming and costly.

DTIs prediction implies determining potential associations among chemical substances and targets in proteins within the human body. In recent years, considerable research efforts have focused on predicting DTIs through computational methodologies, which serve as valuable complements to in vivo validation and significantly reduce both time and cost. Identifying small-molecule targets not only facilitates drug safety evaluation but also accelerates the discovery of novel therapeutic opportunities. Advances in biotechnology have enabled the characterization of a wide range of features associated with drugs and their targets from multiple perspectives, thereby generating massive and heterogeneous datasets. This wealth of data provides a robust foundation for exploring DTIs. Consequently, various Artificial Intelligence (AI) algorithms have been introduced to improve the accuracy and efficiency of pharmacological target prediction.

Among these, deep learning approaches have gained significant traction in the pharmaceutical industry. Coupled with infinite scalability, storage, reliability, safety, saving money as well as maximizing the therapeutic effects of drugs. This expansion has facilitated the access, integration, and structuring of large-scale datasets, encompassing medical imaging, text-based data, authentication, and additional data from gadgets that are portable gadgets, assessment details, and multidimensional data [35]. The influence of Artificial Intelligence on pharmacological development can be stated as innovative because of its ability to eliminate traditional drug design timelines, along with broadening the present drug discovery scale [5]. There are four substantial interests of AI in drug development. The first one is Objectivity, which implies that the development process of drugs using AI is not influenced by presumptions, a prior understanding, or individual passions that could have an immediate influence on the outcomes of development. The second one is constant Progression that enhances the use of cutting-edge biology and computing technology. This process is always growing, with increased levels of creativity and a decrease in cost for AI tools. The third one is higher predictivity. AI algorithms’ heightened aptitude for prediction has a positive impact on the process of determining significant interactions.

The main contributions of this mode are summarized as follows:

1. A novel hybrid model (HDTI-IC50) is proposed to predict the half-maximal inhibitory concentration (IC50) values of p53 inhibitors.
2. The model sequentially integrates Graph Convolutional Networks (GCNs) and Graph Attention Networks (GATs) to effectively capture both local structural patterns and long-range molecular dependencies.
3. A global pooling mechanism combining Global Max Pooling (GMP) and Global Average Pooling (GAP) is applied to aggregate node-level features into a unified graph-level representation.
4. The proposed approach leverages the complementary strengths of GCNs and GATs, enabling a more accurate and interpretable representation of drug–target interactions.
5. Extensive experiments conducted on the Genomics of Drug Sensitivity in Cancer (GDSC) dataset demonstrate that the HDTI-IC50 model outperforms existing DTI models in terms of MAE, RMSE, and R^2^, confirming its effectiveness and robustness.

The rest of this article is structured accordingly. Sect. "Preliminaries" represents the theoretical foundations, including drug–target interaction principles, the p53 protein, and graph neural networks. Sect. "Related work" surveys the existing literature and discusses prior contributions in this domain. Sect. "Proposed model" details the proposed framework, HDTI-IC50, which is designed to enhance predictive performance. Sect. "Experimental results" presents the experimental evaluation along with the key findings. Lastly, Sect. “Conclusion and future work” wraps up the study and highlights promising avenues for further investigation.

## Preliminaries

This section briefly explains the concept of drug-target interactions, its fundamental principles, and the p53 gene, before transitioning to the principles of graph neural networks.

### Drug–target interaction and p53

A target is an internal molecular structure, typically a protein, that is closely associated with a disease process and can be modulated by drugs to achieve the desired therapeutic effect. Drugs work on four different kinds of targets, which include receptors, ion channels, enzymes, as well as carrier molecules. Most medications are proficient in all four scenarios because they engage specific target proteins [3].

The inaugural phase of the discovery of drugs begins with target identification and progresses through lead optimization in later stages. Several sources, including research conducted by universities, experimental medicine, and the business industry, contribute to the selection of an appropriate disease target by determining the purpose of a potential target for therapy (gene/protein) and its significance in the disease. The pharmaceutical business and several research organizations begin with the identification of a suitable biological target for developing approved medications. Once the target is identified, its molecular mechanisms are characterized. A good target should be productive, secure, suit medical and business goals, and be "druggable". Discovering reactions between medicines and target proteins is a pivotal phase in the process of finding new drugs. This assists in comprehending the process of illness and detecting unanticipated treatment actions or pharmacological side effects [2].

**Fig. 1:**
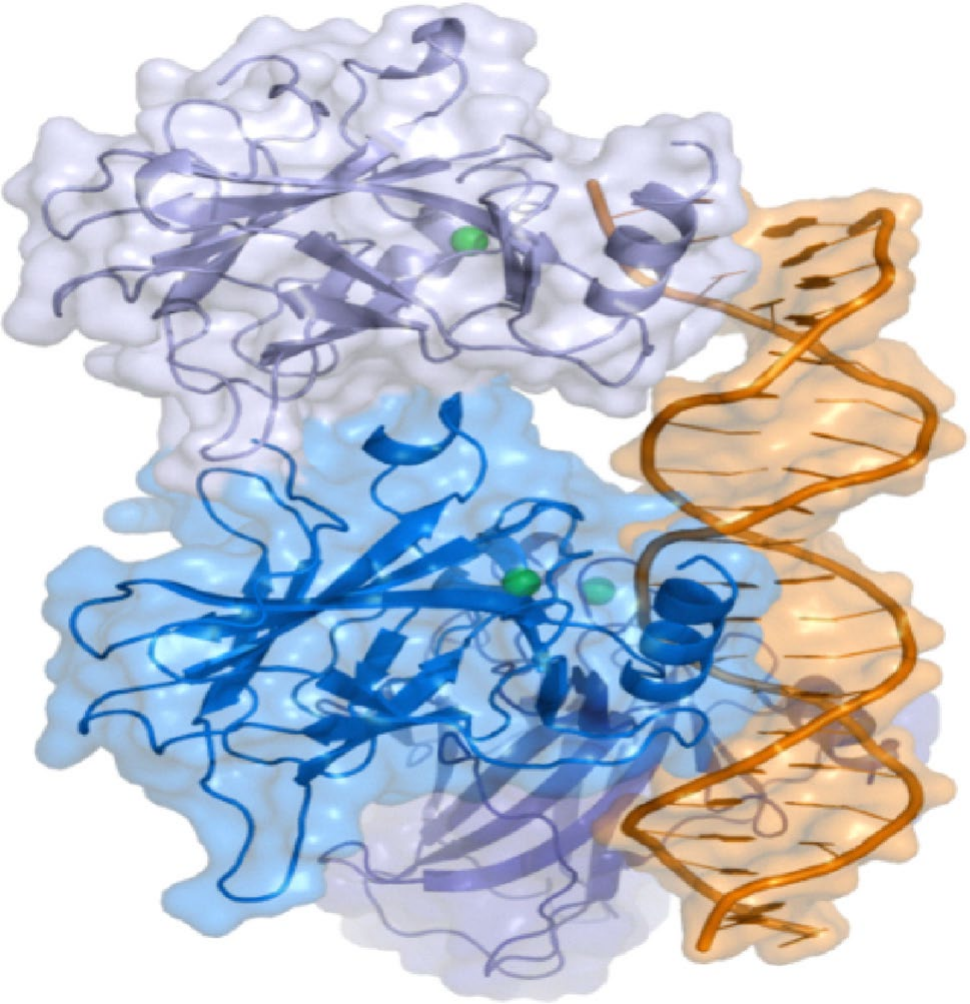
The structure of p53

DTIs could appear in various mechanisms. Competitive inhibitors are drugs that connect themselves to the reaction's effective target site. Another type, known as a lostereroic inhibitor, which operates by modifying the form and architecture of the target to prevent a certain substrate from being detected, hence preventing the biological response of the target [9]. Estimating interactions among drugs and targets has a variety of applications, including drug discovery, repositioning, and adverse effects prediction. Among the many targets in human cancer, one of the most important targets is p53. This target is a tumor inhibitor as well as it is a regulating protein, which is frequently altered in human tumors [34]. In vertebrates, p53 plays a critical role in preventing cancer progression. The tp53 gene encodes the p53 tumor inhibitor protein, which is necessary to preserve genomic stability and cellular DNA integrity. Its primary biological function is to protect the genome from damage, thereby preventing the development of cancer [38]. Figure 1 illustrates the structural composition of the p53 protein.

The p53 protein is found in the cores of cells and binds directly to DNA chromatin. This protein functions as a tumor inhibitor, indicating that it controls the process of cell division by preventing cells from propagating (expanding and splitting) too quickly or in an unpredictable manner. When a cell's DNA is damaged by any stimuli, such as toxic compounds, electromagnetic radiation, or even ultraviolet (UV) rays. The primary functions of this protein are to regulate the cell cycle, induce apoptosis, and promote cellular senescence in response to different stress signals [38]. This protein can determine whether the DNA can be rectified, or the cell will be destroyed by itself, which is called programmed cell death (apoptosis). If the DNA can be rectified, p53 activates further genes to compensate for that damage. If the DNA cannot be rectified, this protein prevents the cell from breaking down and induces apoptosis. p53 aids in the prevention of tumor formation by inhibiting the division of cells with mutant or damaged DNA [40]. Mutations in the tp53 gene are responsible for around fifty per cent of all cancers that occur in humans, including the skin, breast, colon, lung, liver, prostate, and bladder [6].

### Graph neural networks

**Fig. 2:**
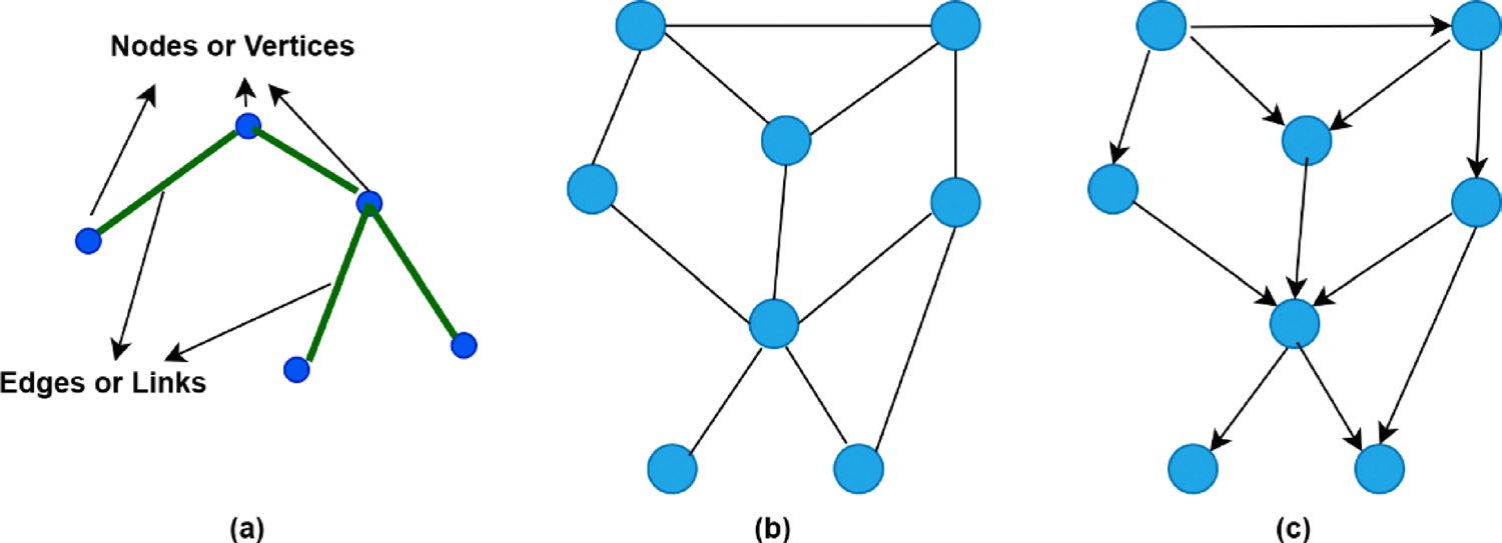
**a** represents the graph structure according to nodes and edges, **b** represents the undirected graphs, **c** represents the directed graph

Graphs are non-Euclidean data structures that imitate data from complex real-life situations where standard forms like images, music, or sequencing text may fail. Brain networks, chemical components, and the classic example of social networks constitute some of the more complex cases [4]. In computational science, a graph is defined as a data structure consisting of a couple of elements: nodes (vertices, V) and edges (E). A graph G is typically referred to as a graph G = (V, E), whereas V represents a collection of nodes that represent the elements of the graph and E represents the edges that determine the relationships between nodes and connect them. This means that G contains a set of vertices or nodes V, along with a suite of edges E. As illustrated in Fig. 2, Nodes and edges can also be coupled with attributes that identify the distinctive features of the entities and the relationships they represent. If nodes have directive relationships, the edges are referred to as directed edges, and the graphs are known as directed graphs. Alternatively, the edges are referred to as undirected edges; additionally, the graph is known as an undirected graph.

Molecules are the core components of a substance, composed of atoms and electrons in three-dimensional space [13]. All the particles interact, but as soon as two atoms are trapped at a constant distance from each other, we call this a covalent bond. The distances between atoms and bonds vary (single or double bonds). It is a widespread abstraction to characterize this 3D object as a graph, regarding nodes as denoting atoms and edges as denoting covalent bonds. GNNs are a type of neural network that applies predictive power as a class of deep learning methods. Deep learning is a powerful algorithm of machine learning that has been comprehensively researched. GNN is learned for rich data structures that are constructed to execute inference on data represented by graphs according to the concept of nodes and edges [41]. GNNs are applied to graphs and provide an easy way to make useful predictions at the three levels of graph structure represent nodes, edges, and the graph. GNNs can do what Convolutional Neural Networks (CNNs) failed to do. GNN architecture's foundational objective is to acquire an embedding using information regarding its surroundings. We can utilize this embedding to help address various types of issues, including node labeling and node-edge prediction [15]. Graph Neural Networks are categorized into three types [28], which are the Recurrent Graph Neural Network, Graph Attention Network, and Graph Convolution Neural Network.

GNNs' core backbone is known as the Message-Passing framework, which became a powerful standard for many years in the era. The Message-Passing Framework is responsible for sharing data between nodes through the process of aggregation and combination of inserting node information along with edge information into node embeddings through the message-passing layers [28]. According to the Message-Passing framework, for every individual node in the graph, there are a couple of operations:

- Aggregation: This process is accomplished by aggregating the information from the node’s neighbors that share an edge with the central node and transforming it to create a representation of the neighborhood [37].
- Combination: This process is accomplished by updating the node information. The outcome of the aggregation is combined with the current value of the node to produce its novel embedding, which is utilized to alter the visualization of every distinct node.

**Fig. 3:**
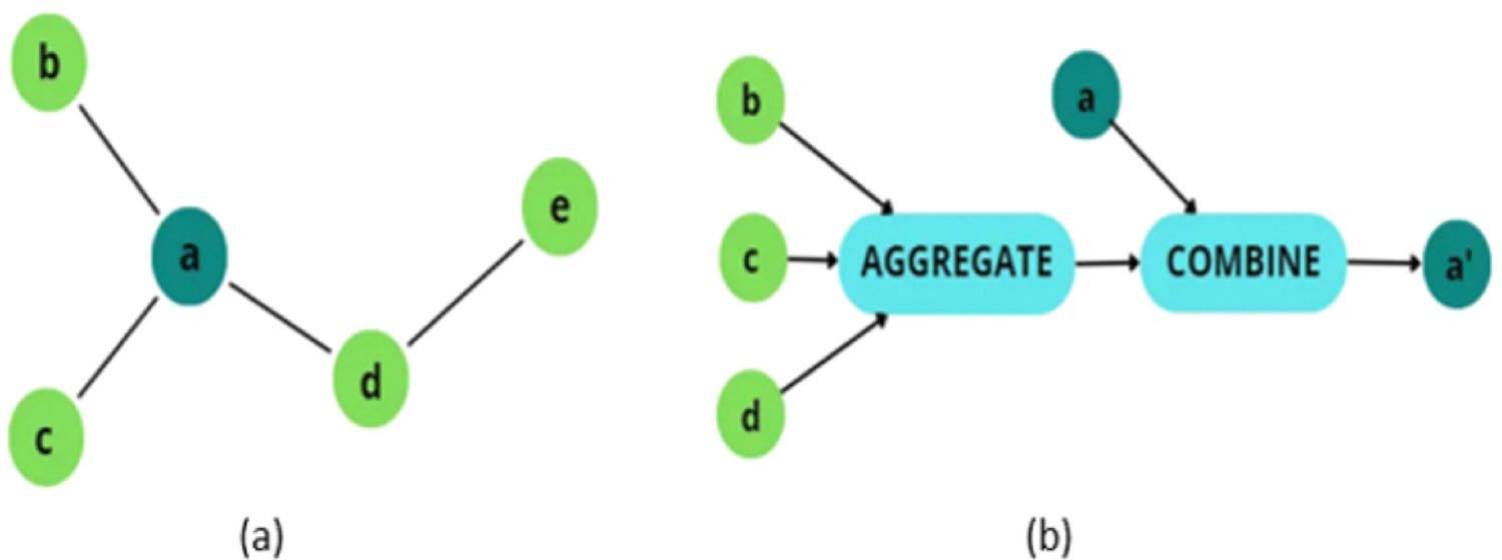
**a** Example of a graph with five nodes. **b** Representation of the aggregation and combination steps from the perspective of the node

As an example, depicted in Fig. 3, if the individual node a is concentrated, the information from its neighbors’ nodes b, c, and d will be passed to an aggregation process, and the current value of node a will be combined along with the result of the previous operation and resulting new value for node a.

## Related work

Within AI models, GNNs are gaining traction as robust artificial neural networks that process and gain information from graph-structured data. Graphs are commonly exploited to depict complex relationships and interactions among many entities. Because of its adaptability, GNNs are increasingly being employed at various stages in the development and discovery of drugs. The identification and confirmation of the target procedure employs GNNs to find prospective therapeutic targets by the examination of gene expression data, protein–protein interaction networks, and other biological data. Furthermore, GNNs can be utilized to anticipate the binding affinity of tiny molecules to protein targets, identify prospective lead compounds, and optimize their chemical properties. Furthermore, GNNs can be exploited to predict small compounds' Absorption, Distribution, Metabolism, Excretion, and Toxicity (ADMET) characteristics, including toxicity, permeability, and solubility. Another potential application for GNNs is Drug-Target Interaction prediction and side effects, which can predict drug interactions with their targets, including protein–ligand binding affinity and specificity. Furthermore, GNNs have been extended to predict drug toxicity, as demonstrated in [20]. It proposed an enhanced GNN architecture that models both indirect interactions between nodes and direct node-to-node relationships within the molecular graph. Their approach achieved superior performance compared with conventional GNN architectures and other machine learning algorithms.

Furthermore, GNNs can be utilized in drug repurposing and repositioning to find new applications for current medications by analyzing their chemical structures, biological activities, and illness connections. Furthermore, GNNs can be employed in de novo medication development to produce novel chemical compounds with desirable features, including binding affinity, selectivity, and pharmacokinetics. Several research reviews have shown that GNNs have greater potential for drug discovery and are outlined by utilizing a variety of datasets, deep learning models, and machine learning algorithms. Jiang et al. [14] suggested a study that contrasted four conventional algorithms with GNN algorithms such as Graph GCN and GAT. Because GNNs can capture intricate chemical characteristics, their performance improved.

Devipriya et al. [7] proposed a model that predicts IC50 for neurodegenerative disorders. This model employed a graph convolutional neural network as a deep learning model, as well as the Deepchem framework for the implementation. The proposed model is evaluated by usage, the UniProt database, and its effectiveness is evaluated through regression measures, such as coefficient of determination (R^2^), mean absolute error (MAE), and root mean square error (RMSE).

Jihye Shin et al. [31] established a comprehensive model dubbed DRPreter (drug response predictor and interpreter), which estimates chemotherapy drug reaction. The model is a regression-based framework designed to anticipate the half-maximal inhibitory concentration (IC50), which serves as a reliable predictor of drug sensitivity. The model is learned and evaluated using the GDSC (Genomics of Drug Sensitivity and Cancer) dataset. Aron Park et al. [26] investigated a model based on ML and deep learning for predicting IC50 values. This model reported that deep learning surpassed machine learning in a variety of circumstances. To enhance prediction performance, they proposed an improved ResNet-based architecture. The ResNet model was employed to provide additional possibilities for gradient adjustments and backpropagation.

Lee et al. [18] developed a comparative model evaluating two CNN architectures—GoogLeNet and AlexNet—alongside the LASSO regression method, as the basis for drug responsiveness classification into three categories. Cell line mutation statuses and drug molecular attributes (including molecular fingerprints) to estimate upper, middle, and lower ranges of IC_50_ values. In addition, we examined the models' treatment responsiveness in breast cancer patients and independent stomach cancer cell lines. The result demonstrated that the CNN models outperformed the ML models in the comparative analyses. According to the research by Patrício et al. [27], a Quantitative Structure–Activity Relationship (QSAR) classification framework was established to forecast tiny compounds' bioactivity targeting the p53 protein, based on empirical molecular descriptors and structural fingerprints. A curated dataset comprising 10,505 compounds was assembled from the ChEMBL, ZINC, and Reaxys databases, with each molecule annotated as either active or inactive according to its potency. To construct predictive models for identifying potential p53 inhibitors, the study employed machine learning techniques, such as CNN algorithms, Random Forest (RF), and Support Vector Machine (SVM).

In parallel, graph-based learning approaches have gained traction because of their capability to depict molecules as topological structures. Kim et al. (2021) presented DrugGCN, a deep learning framework that integrates GCNs with gene expression data to predict drug response. Their method constructs a gene-level interaction graph by combining protein–protein interaction (PPI) networks with transcriptomic profiles and applies feature selection to isolate drug-relevant genes. The GCN subsequently employs localized spectral filtering to learn high order features predictive of drug sensitivity. Furthermore, the authors demonstrated that their GCN model is more effective than other machine learning methods. Furthermore, a GCN-based graph deep learning model incorporating an attention mechanism has been employed to predict synergistic drug combinations [21]. This model can also outperform the ML algorithms. DrugGCN's effectiveness was examined using four GDSC datasets, and it demonstrated accurate prediction regarding the RMSE, MAE, and R^2^. 

Song et al. [32] proposed a MIDTI model for DTI prediction that more effectively integrates diverse similarity data. The method begins by constructing multi-view similarity networks for both drugs and targets and fuses these networks in an unsupervised fashion to obtain integrated embeddings. A deep interactive attention mechanism is then applied. Finally, a multilayer perceptron processes the refined features to predict potential DTIs.

Furthermore, multi-view strategies have also demonstrated strong performance in related tasks, such as synergistic drug combination prediction, as proposed by Monem et al. [22]. Their model integrates multi-view drug feature representation and multi-view cancer cell line features to enhance the prediction of drug combinations.

By leveraging a multimodal fusion strategy alongside adaptive representation learning, MultiCTox addresses a common limitation in earlier cardiotoxicity models that rely on single-modal data. Its design ensures more robust and expressive molecular representations, effectively encapsulates diverse chemical features. This results in improved predictive accuracy for cardiotoxicity outcomes [10].

StackDILI [12] presents a robust computational model for predicting drug-induced liver injury (DILI) that strategically combines diverse molecular representations with a stacking ensemble learning strategy. This model depends on a stacking ensemble consisting of tree-based learners (such as RF, XGBoost, and Histogram-based Gradient Boosting), whose combined outputs enhance both prediction accuracy and interpretability of hepatotoxicity risk. Table 1 briefly summarizes related work studies of using graph neural networks in the drug discovery process.

**Table 1:** Summary of Related Work

Authors	Model/Approach	Technique(s)	Task	Key dataset (s)	Key outcomes/metrics
Jiang et al. [14]	Comparison of GCN and GAT vs. traditional ML	Graph Neural Networks	Drug discovery via chemical structure learning	ESOL, FreeSolv, BBBP	GNNs outperformed traditional algorithms in capturing complex chemical features
Devipriya and Vijaya [7]	GCN for IC_50_ prediction in neurodegenerative diseases	GCN and DeepChem	IC50 prediction	UniProt	Evaluated with RMSE, MAE, and R^2^ metrics
Shin et al. [31]	DRPreter (Drug Response Predictor and Interpreter)	Regression-based deep learning	IC50 prediction (chemo drug sensitivity)	GDSC	Accurate drug sensitivity prediction via IC50
Park et al. [26]	ResNet-enhanced model	Deep Learning (ResNet) vs. Machine Learning	IC50 prediction	GDSC	DL models (ResNet) outperformed ML in various scenarios
Lee and Nam [18]	Comparative study: GoogLeNet, AlexNet, LASSO	CNNs and LASSO regression	IC50 classification (high, medium, low)	GDSC (Breast & stomach cancer datasets)	CNNs outperformed traditional ML models
Patrício et al. [27]	QSAR classification framework for p53 inhibitors	ML (RF, SVM), CNN	Bioactivity prediction (active vs. inactive)	ChEMBL, ZINC, Reaxys	ML models built to predict p53 inhibitor activity
Kim et al. (2021)	DrugGCN	GCN + PPI + gene expression	Drug response prediction	GDSC (4 subsets)	DrugGCN outperformed ML models (metrics: RMSE, PCC, SCC)
Song et al. [32]	Multi-View Similarity Network Fusion Drug–target heterogeneous network	DTIs	Luo’s dataset	Multi-view outperformed traditional ML and DL models	
Feng et al. [10]	Multimodal learning for DTI prediction	Graph-encoder + sequence-encoder + fingerprint- encoder	Cardiotoxicity prediction	(hERG, Nav1.5, Cav1.2)	Multi-model outperforms ML models in cardiotoxicity prediction
Guan et al. (StackDILI [12] )	Stacking ensemble of molecular descriptors	Molecular + genetic-algorithm feature selection + stacking ensemble	DILI prediction	DILIrank	Stacking ensemble outperforms ML models

Existing studies have either focused on general IC50 prediction across diverse compounds or on the binary classification of p53 inhibitors as active or inactive, without providing a unified framework for predicting IC50 values specific to p53 inhibitors. Moreover, many approaches rely solely on traditional molecular descriptors or fingerprints, thereby overlooking the structural and topological information that can be effectively captured through graph-based representations. Additionally, prior studies have generally employed GCNs or GATs independently, without leveraging their complementary strengths. The sequential integration of GCNs, which extract localized structural features, with GATs, which highlight the most informative molecular interactions through attention mechanisms, remains largely unexplored. Furthermore, the explainability of predictions has often been neglected, despite its crucial role in drug discovery applications.

Finally, only limited efforts have been dedicated to the use of GNN-based architecture specifically for p53 inhibitors, even though this protein is a well-established tumor suppressor and one of the most critical drug targets in oncology. This gap highlights the need for a specialized and interpretable predictive model capable of accurately estimating IC50 values for p53 inhibitors by integrating advanced GNN methodologies.

## Proposed model

**Fig. 4:**
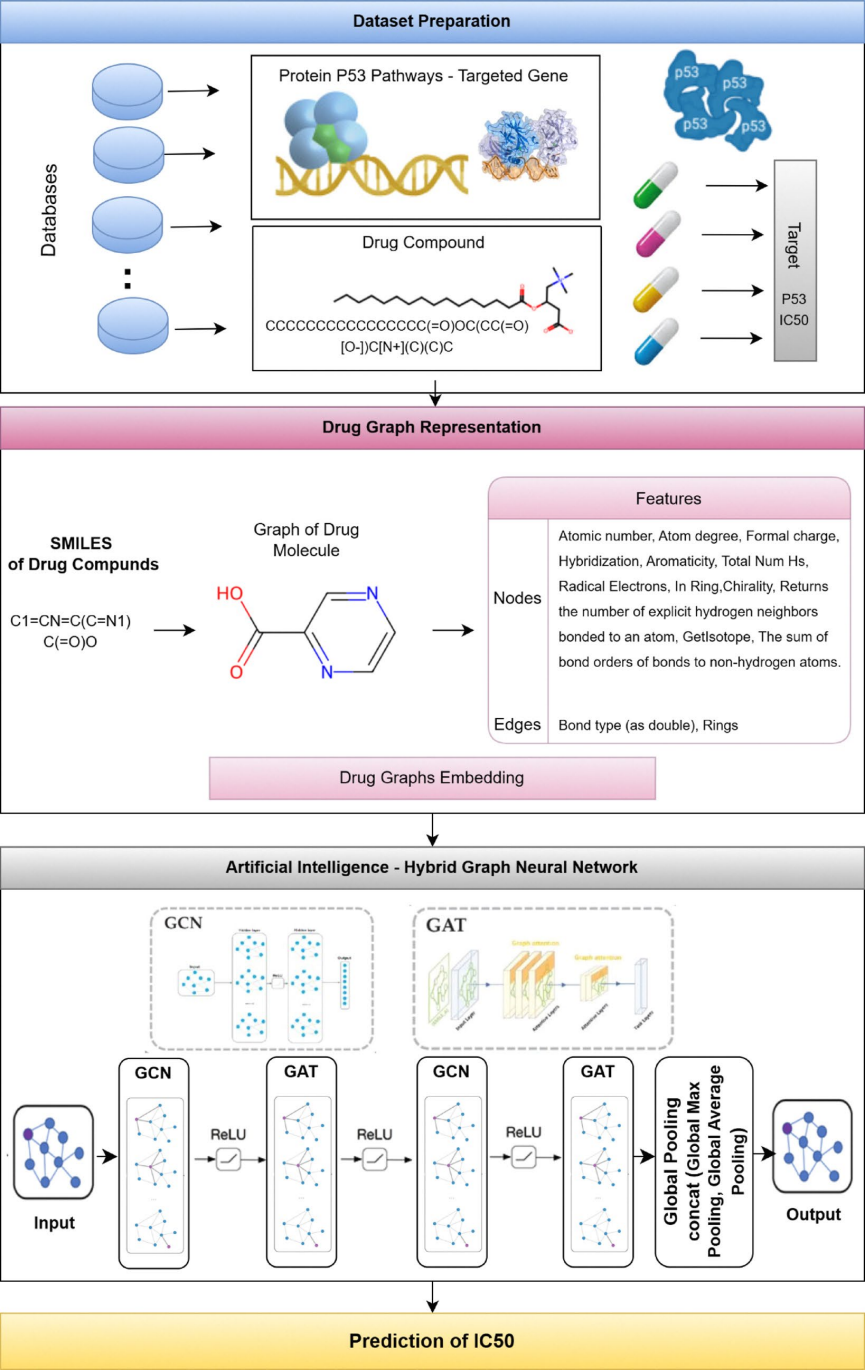
The General framework of the proposed model: HDTI-IC50 Prediction Model

This section presents the proposed Hybrid DTI–IC50 (HDTI-IC50) model, designed to investigate the p53 inhibitors and predict the IC50. The model traverses several phases. The first phase is data preparation. Second is drug-graph representation, followed by the phase of merging the GCN and GAT layers to build the network layers, followed by global pooling, and finally predicting IC50, as illustrated in Fig. 4.

The utilized datasets are described and straightened out, accompanied by subsections explaining each of these phases. The effectiveness of the suggested model is evaluated using evaluation metrics, included MAE, RMSE, and R^2^. The source code is implemented with python 3.10 with torch and the torch geometric package.

### Dataset preparation

The primary dataset used to evaluate the proposed model (HDTI-IC50) is obtained from the Genomics of Drug Sensitivity in Cancer project. This project is a cooperation between the Cancer Genome Research Project at the Wellcome Sanger Institute in the United Kingdom and the Centre for Molecular Therapeutics at the Massachusetts General Hospital Cancer Center in the United States. This cooperation intends to determine biological indicators of cancer that can be employed to examine subgroups of individuals with specific genetic features most likely to respond to cancer therapies. The project involves screening over 1000 biologically designated cancer cell lines from human beings with various anti-cancer drugs, including cytotoxic chemotherapeutics and targeted therapeutics.

The GDSC1000 collection consists of more than 1,000 human tumor cell lines. These cancer cell lines cover the full range of adult and pediatric cancers, both widespread and unusual, encompassing those with epithelium, mesenchymal, and hematopoietic bases. The cell lines are separated according to therapeutic appropriate tissue specifications (GDSC1 and GDSC2) and employ TCGA tumor kind identifiers. Every individual cell line and its metadata are recognized and connected by its own distinctive COSMIC ID. The GDSC1 database information set is extracted through a collaborative effort between the Wellcome Sanger Institute and the collaboration of Massachusetts General Hospital. This dataset comprises a collection of cancer cell lines that are compatible, known as the GDSC1000. The substances were kept in specimens at − 80 °C and underwent no more than 5 freeze–thaw processes. The target pathway p53 is selected with all issue types. The final dataset contains 960 cell lines and 13 types of tissues.

In the data preparation phase, the dataset is initially collected and then preprocessed to remove any ambiguous, repeated, or missing data. To guarantee comprehensive data, records with missing SMILES strings or IC_50_ values or IC50 values that were zero, negative were eliminated before graph building to avoid undefined logarithmic transformations. Subsequently, the inhibitory activity levels IC50 value was transformed into its negative base-10 logarithmic form from IC_50_ to pIC_50_ (− log_10_(IC_50_)) to increase computational reliability and learning converge. To guarantee comprehensive data, records with missing SMILES strings or IC_50_ values were eliminated before graph building. This transformation not only normalizes the scale of activity values but also ensures a direct correlation between the pIC50 and compound potency, where higher pIC50 values correspond to stronger inhibitory activity. dataset was preprocessed to construct chemically meaningful graph representations for all drug molecules. Each compound was represented by its canonical SMILES string. The SMILE (Simplified Molecular Input Line Entry System) structure of the drug is derived from its drug ID. SMILES can describe a three-dimensional chemical structure in a concise text format. Also, they are appropriate for computer interpretation and transformation. It is easy to understand and use for computers and researchers alike [30]. Then, each SMILE was converted into a molecular graph using RDKit. In this representation, atoms correspond to nodes and chemical bonds to edges. For each atom, a comprehensive set of physicochemical and structural descriptors was extracted. Similarly, bond-related features were extracted such as bond type, conjugation, ring membership, and stereochemistry were encoded symmetrically in both bond directions.

This representation is widely adopted in drug discovery research as it facilitates interpretation and model training. As the extracted molecular descriptors have been represented on analogous physical scales, there was no need for additional graph normalization or feature scaling; learning-based normalization was achieved implicitly during model training via batch processing and weight regularization.

### Drug graph generation and featuring

**Fig. 5:**
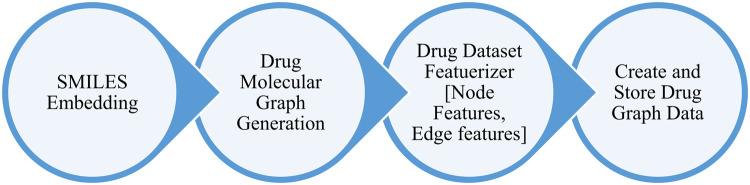
Stages Representation of Drug Graph Generation and Featurizing

In this phase, the drug dataset behaves as the input, while the output is the stored representation of the drug graph network. Initially, molecular graphs are generated for the drug structure. Each drug molecule is then transformed into a graph structure. Finally, the drug graph data is stored for subsequent input into the GNN, as substantiated in Fig. 5.

The SMILES strings are parsed and extracted to construct molecular graphs, where each atom is represented as a node and each chemical bond as an edge connecting the nodes. Table 2 illustrates the drug name, ID, SMILE, and Molecule.

**Table 2:** SMILES embedding and drug molecular graph generation

Drug name	Drug ID	Drug SMILE	Drug molecule
NSC-207895	269	CC(C(= O)NC(CCC(= O)NC(CCCC(C(= O)O)N)C(= O)NC(C)C(= O)NC(C)C(= O)O)C(= O)O)NC(= O)C(C)OC1C(C(OC(C1O)CO)OP(= O)(O)OP(= O)(O)OCC = C(C)CCC = C(C)CCC = C(C)CCC = C(C)CCC = C(C)CCC = C(C)CCC = C(C)CCC = C(C)CCC = C(C)CCC = C(C)CCC = C(C)C)NC(= O)C	
NSC319726	461	CCCCCCCCCCCCCCCC(= O)OC(CC(= O)[O-])C[N +](C)(C)C	
Nutlin-3a (−)	1047	C1 = CN = C(C = N1)C(= O)O	
Serdemetan	1133	C(C(= O)O)S	

#### Atom/Node features

These features represent the individual atoms within a molecule. Some of the key properties and their extraction methods are outlined below.

- **Atomic number**: this feature get the quantity of protons in an atom's kernel, which is distinctive of a chemical substance and determines its location in the periodic table [29].
- **Atom degree**: this feature retrieves the level of every atom in the structure of the molecule. Atom's level is stated as the overall amount of adjacent bond neighbors.
- **Formal charge**: A formal charge (FC) is the charge assigned to an atom in a molecule.
- **Hybridization**: this feature returns the ‘hybridization’ property of an atom. The definition of hybridization is the procedure of merging a pair of orbitals of an atom to generate a novel kind of hybridized orbitals [11].
- **Aromaticity**: Aromaticity can be characterized as a feature of cyclic conjugated cycloalkenes that promotes the long-term stability of a molecule by delocalized electrons with (4N + 2)(4*N* + 2) number of ππ electrons as aromatic (where N*N* is zero or any positive integer).
- **Total Num Hydrogens**: represents the total number of both visible and hidden hydrogens in the individual atom.
- **Radical Electrons**: represents the number of radical electrons for this Atom.
- **Ring Membership**: determines if the elementary particle atom resides inside a ring.
- **Chirality**: this feature retrieves the Molecule that possesses this atom. Returns the atom's monomer information object, if one exists.
- **Explicit Valence**: represents the number of explicit hydrogen neighbors bonded to an atom.
- **Isotopic Mass**: it is the average weight of all isotopes of a given element.
- **Total Valence**: represents the sum of bond orders of bonds to non-hydrogen atoms. It’s the atom's overall valence, including clarified and also implied.

#### Edge features

Representing the bonds or links between molecules. Some of its important properties and methods are explained in this section.

- **Bond Type**: this feature specifies the bond particular sort in the form of a double. For example, 1.0 for SINGLE or 1.5 for AROMATIC, along with 2.0 for DOUBLE.
- **Ring Membership**: this feature indicates if the atom is in a ring.

All node features are appended altogether to constitute a node feature matrix, alongside all bond features are appended on the whole to constitute an edge matrix with [Number of edges, Edge Feature size]. As illustrated in Table 3.

**Table 3:** Atom and Edge Features summarization

**Atom features**	
Atomic Number	Returns the atomic number
Atom Degree	Returns the total number of bonds
Formal Charge	Returns the ‘formal charge’ property
Hybridization	Returns the ‘hybridization’ property
Aromaticity	Returns the ‘aromatic’ property
Total Num Hs	Returns the total number of Hs
Radical Electrons	Returns the number of radical electrons
Ring Membership	Indicates if the atom is in a ring
Chirality	Returns the Molecule that possesses this atom
Explicit Valence	Returns the number of explicit hydrogen neighbors
Isotopic Mass	Returns the is the average weight of all isotopes
Total Valence	Returns the total valence (explicit and implicit)
**Edge features**	
Bond Type	returns the type of the bond
Ring Membership	Indicates if the atom is in a ring

The graph architecture is depicted by the adjacency matrix in the form of an N × N matrix that expresses the nodes that are connected by which edges. Then, a collection of data objects is constructed and stored in the processed_dir. Since storing a large list takes considerable time, the list is combined into a single large Data item. The collated data object concatenates all features into one big data object. All Atom features as well as characteristics of the bond are concatenated onto an individual feature vector for every node in the graph of drug data. Concatenation and weighted sum are two prominent aggregation approaches. They are applied to combine the atom features and bond features. This is not only diminishing the degree of dimensionality of the data but also improving the effectiveness of processing.

The drug graph and adjacency matrix are stored as separate single TensorFlow tensors. This format is the most appropriate for using and feeding graphs into graph neural network models. Finally, in this phase, the drug graph dataset is saved and stored in memory using ‘torch_geometric.data.Dataset’. This roughly corresponds to the notions of the torchvision datasets.

### Artificial intelligence- hybrid graph neural network

The input to this phase of the proposed model consists of the drug's molecule-level adjacency matrix and the feature matrix. The adjacency matrix is a square matrix that is composed of equal-sized rows and columns, such that each row and each column represent an atom in the molecule. Each entry in the matrix indicates the presence or absence of a chemical bond between two atoms: a value of 1 denotes the existence of a bond (edge), while a value of 0 signifies no direct connection.

**Fig. 6:**
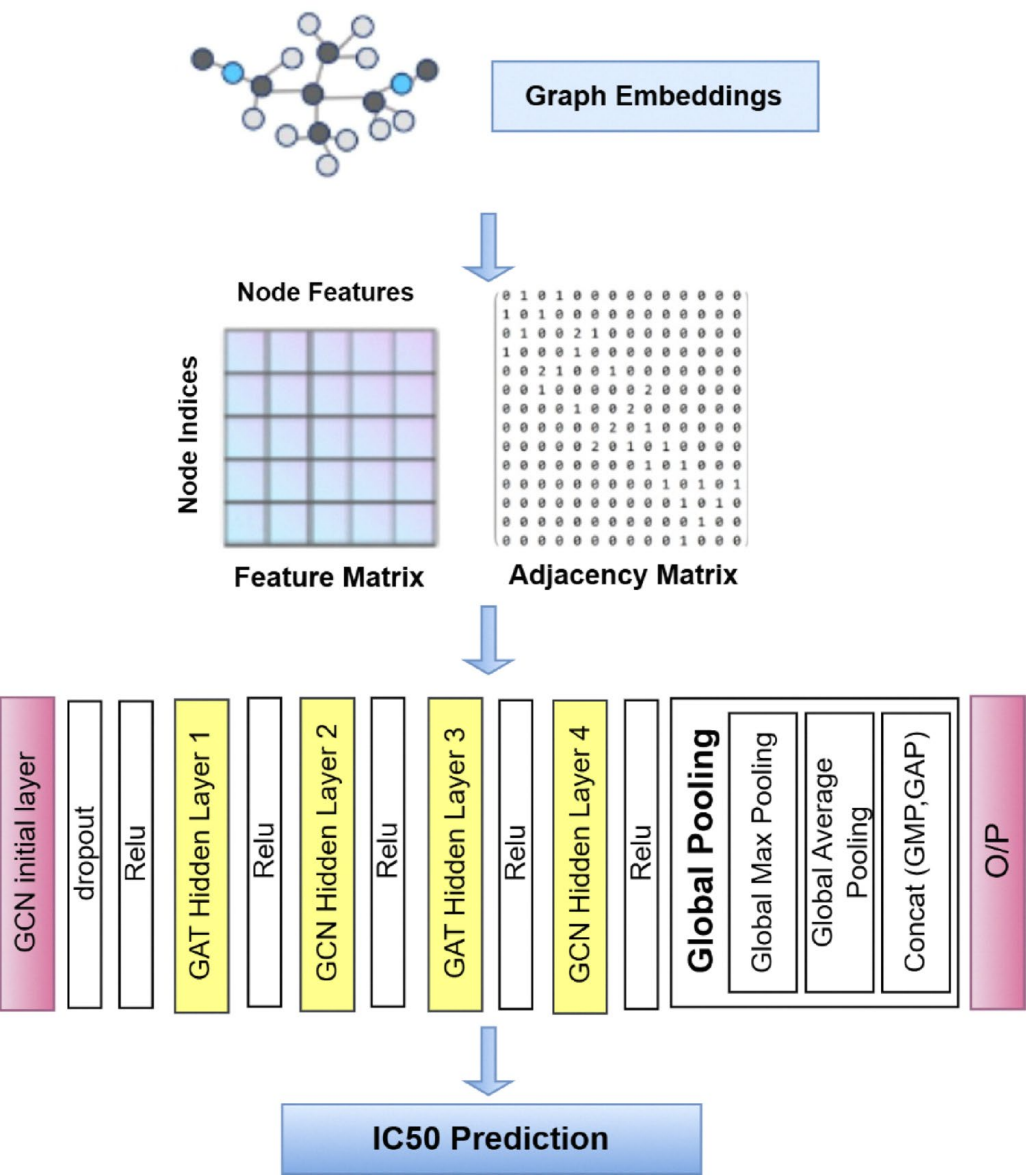
The hybrid convolution network of HDTI-IC50 model

Graph Neural Networks, including GCNs and GATs, are especially well-suited for handling non-Euclidean, graph-structured data, enabling efficient and accurate modeling of molecular structures and their complex interactions. The proposed model (HDTI-IC50) integrates GCN and GAT, followed by global pooling with combined aggregation strategies. Figure 6 represents the hybrid model architecture that includes:

- An initial graph convolutional layer.
- Five hidden layers integrating GCN and GAT with ReLU activation.
- Global pooling.
- Prediction of IC50 values.

The main concern of GCNs is the adaptation of the convolutional process to graphs by aggregating features from a node’s neighbors in a uniform way [25]. This process calculates weighted averages of neighboring node features, outcoming the central node representations. In these layers, GCNs gain feature abstraction [33]. Each GCN layer performs a combination of:

- Graph convolution
- Linear transformation
- Nonlinear activation function

These operations form a single network layer. One or more layers can be amalgamated to create a whole GCN, and its node embedding can be written in this formula.

- Initialize the activation units as in Eq. (1):
  1 $${h}_{v}^{0}=f\left({X}_{v}\right),$$
  where ${X}_{v}$ represents the input feature vector of node v, and ${h}_{v}^{0}$​ denotes the initial embedding of node v, which is typically initialized directly from its input features before being updated in subsequent layers.
- For every layer in the network, the node feature map is updated according to Eq. (2).
  2 $${h}_{v}^{k}=f\left({W}_{k}{\sum }_{u\in N(j)} \frac{1}{|N(v)|}{h}_{u}^{k-1}+{B}_{k}{h}_{v}^{k-1}\right),\forall k\in \left\{1,\dots ,K\right\},$$
  Where ${h}_{v}^{k}$ is embedding feature map of node $v$ embedding after $k$ layers of neighborhood aggregation and ${W}_{k}$ represents the learnable weight matrix of the k-th layer. The term ${\sum }_{u\in N(j)} \frac{1}{|N(v)|}{h}_{u}^{k-1}$ represents the neighbors' representations and ${B}_{k}{h}_{v}^{k-1}$ represents the preceding layer $k-1$ embedding and representation of node $v$ amplified by an element of bias.
  Moreover, B_K_ is an adaptable matrix and is considered a self-loop that activates the v node, and $f$ is not a linear activating function, for instance, the ReLU activation function.
- At the final layer, the final embedding representation of node v after K layers ‘$Zv$’ is as represented in Eq. (3).
  3 $$Zv=\left({h}_{v}^{k}\right),$$
  where ${h}_{v}^{k}$ is the output embedding of node v at the last GCN layer

Graphs are frequently illustrated with an adjacency matrix (A). If a graph involves nodes, its dimension is (n × n). Each node in a network has a set of features (f), and its feature matrix (X) has dimension (n × f). GCN is effective on homogeneous graphs when all neighbors' contributions are equally important.

GATs represent an advanced GNN architecture that utilizes self-attention mechanisms to weigh the significance of every node's feature with regard to those of its neighboring nodes. Each GAT layer performs:

1. Feature linear transformation: Each node's features are first linearly transformed.
2. Attention Coefficient Calculation: Self-attention mechanism computes attention coefficients.
3. Feature Aggregation: Each node aggregates its neighbors’ features, weighted by their learned attention scores, and results in a new feature vector that pays more attention to important neighbors.
4. Multi-head attention: Implements the attention mechanism concurrently across multiple heads to enhance model stability and capture diverse feature representations. Then either concatenate the outputs for hidden layers or average them for the final layer.

The output of the GAT is a collection of node representations that capture the graph's topological structure and associated node or edge attributes [36].

GCNs and GATs have different strengths in learning node representations. GCNs learn local structural information of molecular graphs through spectral-based convolution, which ensures smooth feature propagation across nodes. Also, they are more suitable for homophily (i.e., nodes that share the same attributes are much more probable to form connections). However, GCN treats all neighbors with equal importance, which may overlook the varying relevance of different atoms or bonds. While GATs learn long-range dependencies and are more suitable for heterophily (i.e., nodes with different features are more likely to be connected) [17]. GATs introduce an attention mechanism that assigns learnable weights to neighboring nodes, allowing the model to focus more on the most informative atoms and substructures in the molecule. The proposed model can handle both cases by sequentially incorporating GAT layers after the GCN layers. This sequential combination enables the model to first extract global structural features (via GCN) and then refine them with localized, context-dependent importance weighting (via GAT), leading to more accurate and interpretable predictions.

The proposed model (HDTI-IC50) starts with an initial convolution layer, followed by a sequential stacking of GCNs layers and GATs layers on top of each other. Each hidden layer uses the ReLU activation function, followed by a global pooling process, while closing output is produced via a fully connected linear layer. In the GCN hidden layers, the attributes are agglomerated from neighboring nodes via a propagation mechanism to generate the subsequent layer’s attributes. This aggregation process enables the network to learn progressively more complex higher-level representations and hierarchical structural information. The subsequent GAT hidden layers convert the initial node features into more abstract and higher-level representations that capture the complex relationships between nodes and compute attention coefficients that represent the relevance of a node's features in comparison to those of its surrounding neighbors. A non-linear activation function is applied after each graph attention layer, introducing non-linearity and enhancing the model’s capability, allowing it to discover more complex, non-trivial relationships between nodes across the graph structure.

After processing through the GCN and GAT layers, the proposed model (HDTI-IC5) performs a global pooling mechanism by combining aggregation strategies to produce a fixed-size graph-level representation. It captures both localized and holistic characteristics of the graph structure. Specifically, the model applies a combination of Global Max Pooling (GMP) and Global Average Pooling (GAP) to accumulate node-level features into a single vector per graph. Through the GMP operation, it computes the maximum value across all nodes within each graph for each feature. This allows the network to capture the most prominent activations for each feature dimension. Through the GAP operation, it computes the mean value of the node features within each graph, preserving the overall distribution and contextual information. Then, the outputs of GMP and GAP are concatenated along the feature dimension (dim = 1) into a single vector per graph as follows in Eq. (4):

4 $${\text{Graph}}\,\, {\text{Representation}} = {\text{Concat}}\left( {{\text{GMP}}\left( {\text{H}} \right),{\text{GAP}}\left( {\text{H}} \right)} \right)$$

where H represents the matrix of hidden node features and Concat represents the concatenation operation.

By stacking both pooling strategies, the model ensures that the graph-level embedding retains both the extreme (max) and average feature activations, leading to a richer and more informative representation for the final prediction.

Finally, the combined graph representation is transmitted via a linear output layer that is fully linked, which predicts the IC50 value for each drug-target pair. This architectural design allows the HDTI-IC50 model to efficiently handle both local and global structural patterns in drug molecular graphs, enhancing its predictive accuracy for p53 inhibitor activities.

### Model sensitivity and optimization factors

Model performance is sensitive to hyperparameters. The factors that affect the model's performance include dropout value, number of epochs, number of hidden layers, learning rate, and embedding size. Proper tuning of certain control parameters is crucial for ensuring the best performance. The collection of data is considered a key component that influences the choice of parameters. HDTI-IC50 Hyperparameter measurements are indicated in Table 4.

**Table 4:** HDTI-IC50 Hyperparameter values setting

Hyperparameter	Value
Embedding size	64
Epochs	1000, 700, 500, 200, 100
Learning rate	0.01, 0.0005
Number of parameters	21,761
Optimizer	Adam
GAT2vConv, GCNConv hidden layers	5 hidden layers
Linear output	(I/P 128, O/P 1)

Ultimately, the HDTI-IC50 prediction model was successfully constructed utilizing GCN and GAT via transient the GDSC1 trained set of data, which somewhat includes the IC50 measurements of active medicinal compounds.

## Experimental results

### Evaluation measures

The DTI-IC50 prediction model's effectiveness is assessed by applying a total of three evaluation metrics, namely RMSE, MAE, and R^2^ score. RMSE calculates the square root of the variations among the results that were received and envisaged at the second moment. RMSE is an ideal alternative for evaluating the effectiveness of models focused on predictive tasks. It seizes a sequence of actual values along with a list of anticipated values, then generates the value of RMSE. The formulation is as follows in Eq. (5):

5 $$\text{RMSE}=\sqrt{\frac{{\sum }_{\text{i}=1}^{\text{n}} {\left({\text{y}}_{\text{Ob}}^{\text{i}}-{\text{y}}_{\text{Pd}}^{\text{i}}\right)}^{2}}{\text{n}}},$$

where n is the total number of samples, *y*_*Ob*_ represents the observed (true) value of the ith sample, and *y*_*Pd*_ represents the predicted value of the ith sample.

Furthermore, MAE is a measure that determines the mean size of the utter faults between the numbers that were anticipated and those that were observed. ${R}^{2}$ depicts the degree of proficiency in the procedure for predicting.

Finally, R^2^ scores are produced by taking a collection of authentic values (*y*_*Ob*_), as well as a collection of anticipated ones (*y*_*Pd*_), and computing the level of coincidence between them. It is formulated as in Eq. (6):

6 $${R}^{2}=1-\frac{RMS{E}^{2}}{\mathit{Var}\left({y}_{Ob}\right)},$$

where

7 $$Var\left({y}_{Ob}\right)={\sum }_{i=1}^{n} {\left({y}_{Ob}^{i}-{\bar{y}}_{Ob}\right)}^{2}.$$

### Results and discussion

The following subsection presents the experimental outcomes of the proposed HDTI-IC50 model. Table 5 demonstrates the output measures of MAE, RMSE, and R^2^ with the parameters of learning rate [0.01] and 5 hybrid hidden layers. The stacked hidden layers comprise three graph attention layers, two convolutional layers, an initial input convolution layer, and a final linear output layer. The learning rate regulates the extent to which the neural network weights move during optimized performance, while decreasing the loss function. With an epoch count of 100, the value of MAE is 0.16, RMSE is 0.23, and R^2^ is 0.3. With 200 epochs, the value of MAE is 0.15, RMSE is 0.22, and R^2^ is 0.19. When trained for 500 epochs, the value of MAE is 0.16, RMSE is 0.2, and R^2^ is 0.8. However, increasing the epoch to 700 epochs, the value of MAE is 0.17, RMSE is 0.22, and R^2^ is 0.4.

**Table 5:** MAE, RMSE, and R^2^ for HDTI-IC50 with the parameters of learning rate [0.01] and 5 hidden layers

	Learning rate	MAE	RMSE	R^2^
100 Epochs	0.01	0.16	0.23	0.3
200 Epochs	0.01	0.15	0.22	0.19
500 Epochs	0.01	0.16	0.2	0.8
700 Epochs	0.01	0.17	0.22	0.4

**Fig. 7:**
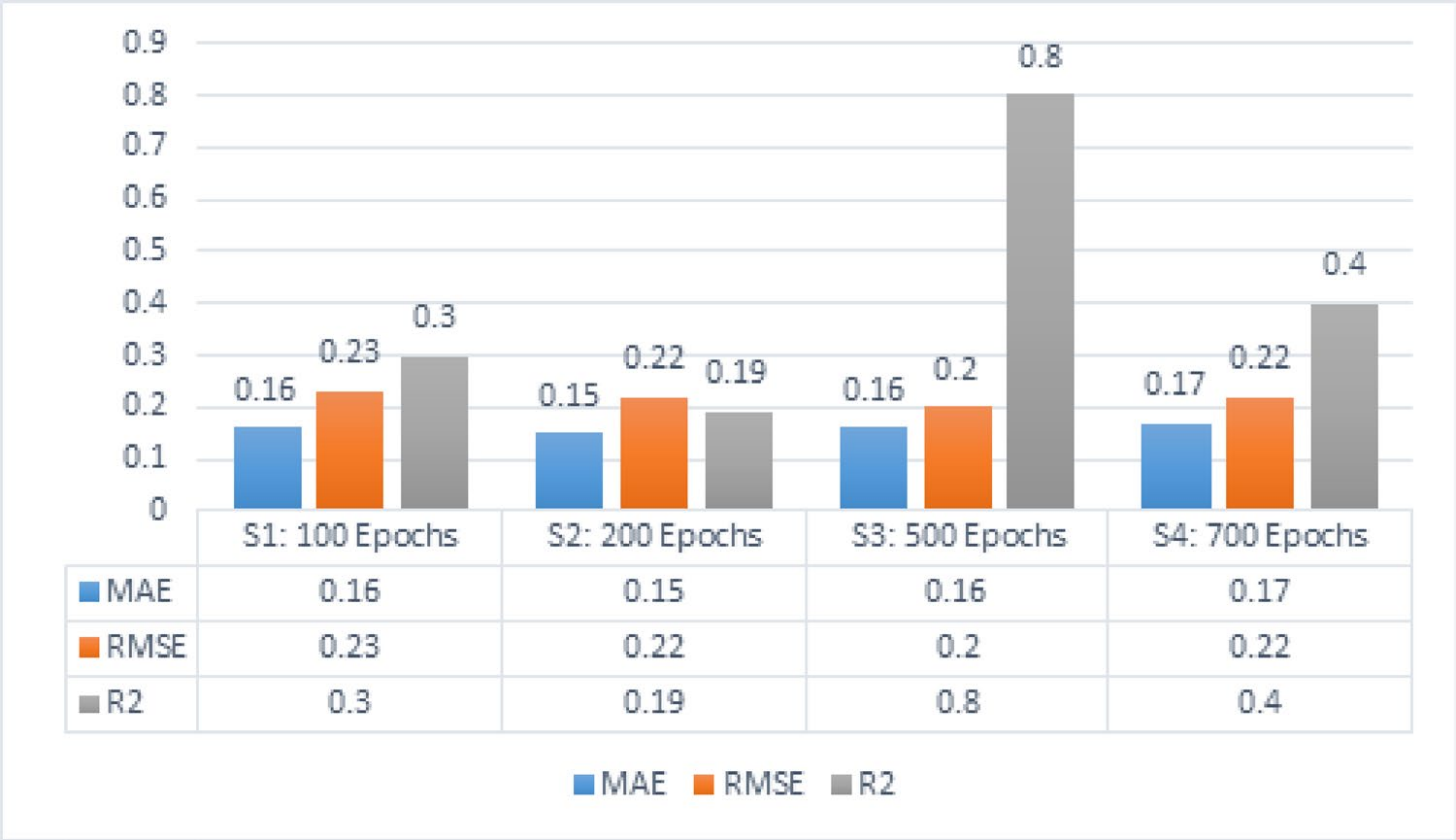
MAE, RMSE, R^2^ for HDTI-IC50 using 5 hidden layers and learning rate 0.01

Figure 7 depicts the results for five hidden hybrid layers with a dropout rate equals 0.2 and a learning rate equals 0.01. Series 1 represents 100 epochs that gained the outcomes of MAE, RMSE, as well as R^2^, respectively, 0.16, 0.23, and 0.3. Series 2 represents 200 epochs that gained the outcomes of MAE, RMSE, alongside R^2^, respectively 0.15, 0.22, and 0.19. Series 3 represents 500 epochs that gained the scores of MAE, RMSE, alongside R^2^, respectively 0.16, 0.2, and 0.8. Series 4 represents 700 epochs that gained the scores of MAE, RMSE, alongside R^2^, respectively 0.17, 0.22, and 0.4.

Table 6 presents the outcomes of MAE, RMSE, and R^2^ with the parameters of learning rate [0.005] and 5 hidden layers. With an epoch count of 300, the scores of MAE equal 0.13, RMSE is 0.17, and R^2^ is 0.6. With 500 epochs, the value of MAE is 0.16, RMSE is 0.19, and R^2^ is 0.8.

**Table 6:** The values of MAE, RMSE, and R^2^ for HDTI-IC50 with the parameters of learning rate [0.005] and 5 hidden layers

	Learning rate	MAE	RMSE	R^2^
S1: 300 Epochs	0.005	0.13	0.17	0.6
S2: 500 Epochs	0.005	0.16	0.19	0.8

**Fig. 8:**
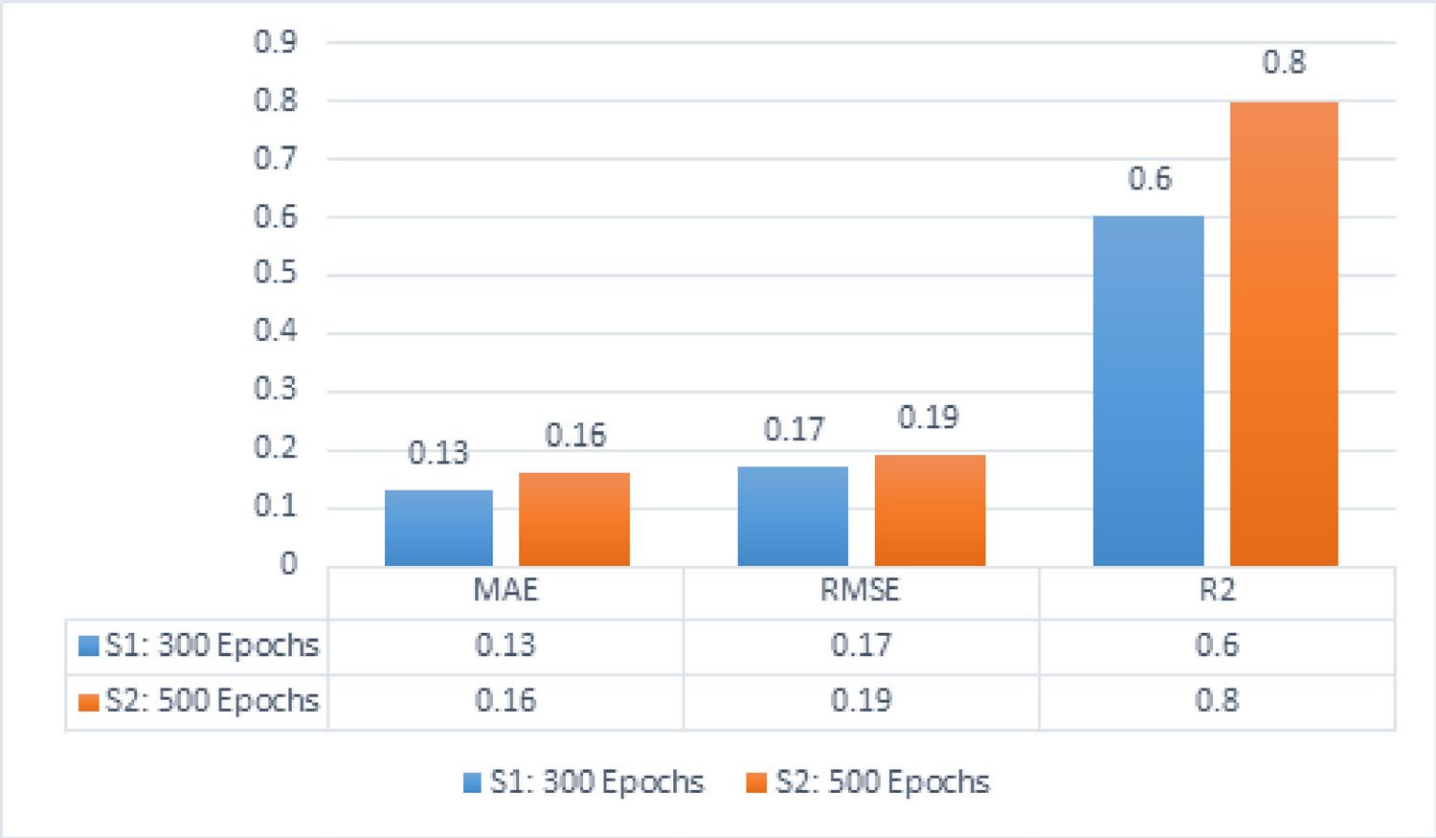
MAE, RMSE, R^2^ for HDTI-IC50 using 5 hidden layers and learning rate 0.005

Figure 8 depicts the results for five hidden layers utilizing a dropout value of 0.2 and a learning rate of 0.005. Series 1 represents the values for a number of Epochs, 300, that gained the outcomes of MAE, RMSE, as well as R^2^, which respectively are 0.13, 0.17, and 0.6. Series 2 represents 500 epochs that gained the outcomes of MAE, RMSE, alongside R^2^, which respectively comprise 0.16, 0.16, and 0.8.

### Inference time and computational efficiency

Inference time refers to the duration required by a trained model to perform a forward pass and generate predictions for a given input, without any parameter updates or gradient computations. It is a crucial indicator for assessing computational performance and real-time applicability of machine learning models, particularly in deployment scenarios where latency is critical. To evaluate the computational efficiency of the model (HDTI-IC50), the average inference time per forward pass by using batched graphs from torch_geometric.data is evaluated.

The experiment is conducted using Torch version 2.1.0 + cpu and PyTorch Geometric version 2.3.1 on a system equipped with [Intel Core i7 processor with 8 GB of RAM]. The inference time over several independent runs using the same graph input is measured. Each measurement captures the duration of a single forward pass. The average inference time is computed as follows in Eq. (8):

8 $$\text{Average Inference Time}=\frac{1}{N}{\sum }_{i=1}^{N}\left({t}_{i}^{\text{end}}-{t}_{i}^{\text{start}}\right),$$

where N denotes the count of runs and ${t}_{i}^{\text{end}}, {t}_{i}^{\text{start}}$​ denote the timestamps after and before the ith forward pass, accordingly. The findings demonstrate that the proposed model (HDTI-IC50) attains an average inference time of 8.12 s per 100 number of epochs, 7.52 s per 200 number of epochs, 7.76 s per 500 number of epochs, and 7.70 s per 700 number of epochs with a learning rate of 0.01 and five hidden layers as shown in Table 7.

**Table 7:** Inference time with learning rate 0.01and five hidden layers

Inference time with learning rate 0.01 and five hidden layers	
No. of epochs	Inference time
100	8.12 s
200	7.52 s
500	7.76 s
700	7.70 s

For five hidden layers and a learning rate of 0.005, the proposed model (HDTI-IC50) achieves an average inference time of 8.66 s per 300 epochs and 7.70 s per 500 epochs, as shown in Table 8.

**Table 8:** Inference time for 5 hidden layers and learning rate 0.005

Inference time for learning rate 0.005 and five hidden layers	
No. of runs	Inference time
300	8.66 s
500	7.70 s

The models’ inference time values demonstrate their suitability for real-time or large-scale processing tasks.

### Cross validation and model robustness

**Fig. 9:**
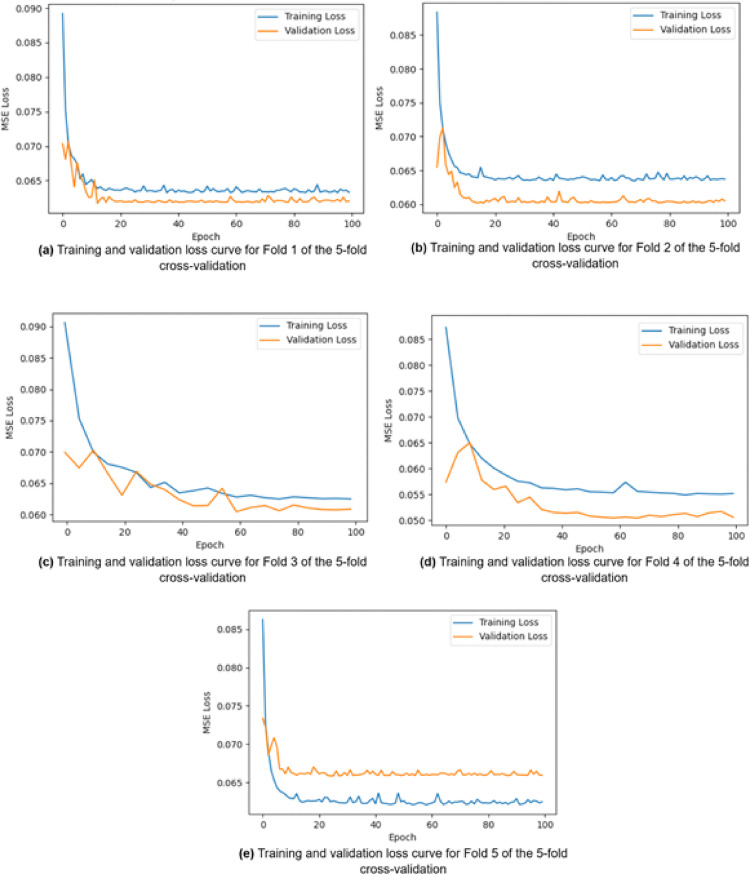
Training and validation loss curves across five folds of cross-validation for the HDTI-IC50 model

Cross-validation is a technique for determining how well a machine learning model works on unknown data while avoiding overfitting. A fivefold cross-validation approach was deployed to guarantee the robustness and generalizability of the suggested HDTI-IC50 model. The findings were averaged over all folds, with 80% of the data utilized for training and 20% for validation in each fold. With an average MAE of 0.174 ± 0.005, RMSE of 0.251 ± 0.015, and R^2^ of 0.14 ± 0.701, the model demonstrated coherent performance through several data segments. To further establish statistical reliability, 95% confidence intervals were calculated. A 95% confidence interval (CI) is a set of values that are 95% probable to include the real population parameter. This indicates that if you execute the sampling procedure several times, about 95% of the confidence intervals generated will contain the population mean. 95% confidence intervals were evaluated for each metric such that MAE is 0.174 ± 0.007, RMSE is 0.251 ± 0.020 and R^2^ is 0.15 ± 0.701. Figure 9 illustrates the training and validation loss trajectories for each fold during the fivefold cross-validation process. The consistent convergence patterns across folds indicate the stability and robustness of the HDTI-IC50 model, confirming its ability to generalize effectively across different data partitions. Table 9. Represents the five-fold cross-validation performance of the proposed HDTI-IC50 model include MAE, RMSE, R^2^, mean ± SD, and 95% confidence intervals.

**Table 9:** Five-fold cross-validation performance of the proposed HDTI-IC50 model include MAE, RMSE, R^2^, mean ± SD, and 95% confidence intervals

Fold	MAE	RMSE	R^2^
1	0.1712	0.249374643	0.6466
2	0.1733	0.247822276	0.8385
3	0.1727	0.270643678	0.7252
4	0.1686	0.22667097	0.8912
5	0.1792	0.25802879	0.5105
Mean ± SD	0.174 ± 0.005	0.251 ± 0.015	0.143 ± 0.722
95% CI	0.1734 ± 0.007	0.251 ± 0.020	0.15 ± 0.701

### Overfitting evaluation

To assess the possibility of overfitting in the proposed HDTI-IC50 model, a systematically training and validation losses is tracked throughout epochs. The loss curves retained its stability, showing that the model acquired significant patterns from the training data rather than rote-learning it. Furthermore, regularized dropout rate (*p* = 0.3) was utilized in the GCN and GATv2 layers to improve generalization. In addition, a fivefold cross-validation technique was used to ensure the model's resilience and avoid overfitting. The model's consistent performance and capacity to generalize to new data are demonstrated by the minimal standard deviation in MAE, RMSE, and R^2^ values across folds.

### Comparative analysis

The following subsection covers a comparative evaluation of the HDTI-IC50 model’s performance against standard basic architectures, GCN and GAT**,** separately. According to the GCN model, Table 10 demonstrates the output measures of MAE, RMSE, and R^2^ based on a learning rate of 0.01 and 5 hidden layers. With an epoch count of 100, the value of MAE is 0.35, RMSE is 0.5, and R^2^ is 0.24. With epochs of 200, the value of MAE is 0.25, RMSE is 0.4, and R^2^ is 0.1. With epochs of 300, the value of MAE is 0.42, RMSE is 0.5, and R^2^ is 0.13. With epochs of 500, the value of MAE is 0.23, RMSE is 0.4, and R^2^ is 0.12. With 700 epochs, the value of MAE is 0.24, RMSE is 0.3, and R^2^ is 0.27.

**Table 10:** MAE, RMSE, and R2 for GCN with the parameters of learning rate [0.01] and 5 hidden layers

	MAE	RMSE	R^2^
S1: 100 Epochs	0.35	0.5	0.24
S2: 200 Epochs	0.25	0.4	0.1
S3: 300 Epochs	0.42	0.5	0.13
S4: 500 Epochs	0.23	0.4	0.12
S5: 700 Epochs	0.24	0.3	0.27

**Fig. 10:**
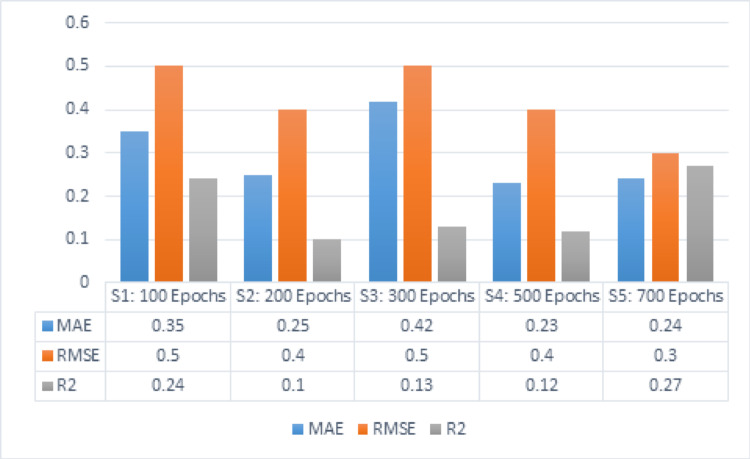
MAE, RMSE, R2 for GCN using 5 hidden layers and learning rate 0.01

Figure 10 MAE, RMSE, R^2^ for GCN using 5 hidden layers and learning rate 0.01. depicts the results for five hidden layers with a dropout value of 0.2 and a learning rate of 0.01. Series 1 represents 100 epochs that gained the outcomes of MAE, RMSE, as well as R^2^, respectively, 0.35, 0.5, and 0.24. Series 2 represents 200 epochs that gained the outcomes of MAE, RMSE, alongside R^2^, respectively 0.25, 0.4 as well as 0.1. Series 3 represents 300 epochs that gained the scores of MAE, RMSE, alongside R^2^, respectively 0.42, 0.5, and 0.13. Series 4 represents 500 epochs that gained the scores of MAE, RMSE, alongside R^2^, respectively 0.23, 0.4, and 0.12. Series 5 represents 700 epochs that gained the scores of MAE, RMSE, alongside R^2^, respectively 0.24, 0.3, and 0.27.

According to the (GAT) model, Table 11 demonstrates the output measures of MAE, RMSE, and R^2^ with the learning rate [0.01] and 5 hidden layers. With an epoch count of 100, the value of MAE is 0.5, RMSE is 0.5, and R^2^ is 0.1. With epochs of 200, the value of MAE is 0.29, RMSE is 0.04, and R^2^ is 0.15. With 300 epochs, the value of MAE is 0.48, RMSE is 0.45, and R^2^ is 0.12. With 500 epochs, the value of MAE is 0.20, RMSE is 0.4, and R^2^ is 0.35. Increasing epochs to 700, the value of MAE is 0.4, RMSE is 0.3, and R^2^ is 0.4.

**Table 11:** MAE, RMSE, and R^2^ for GAT with the parameters of learning rate [0.01] and 5 hidden layers

	MAE	RMSE	R^2^
S1:100 Epochs	0.5	0.5	0.1
S2: 200 Epochs	0.29	0.4	0.15
S3: 300 Epochs	0.48	0.45	0.12
S4: 500 Epochs	0.20	0.4	0.35
S5: 700 Epochs	0.4	0.3	0.4

**Fig. 11:**
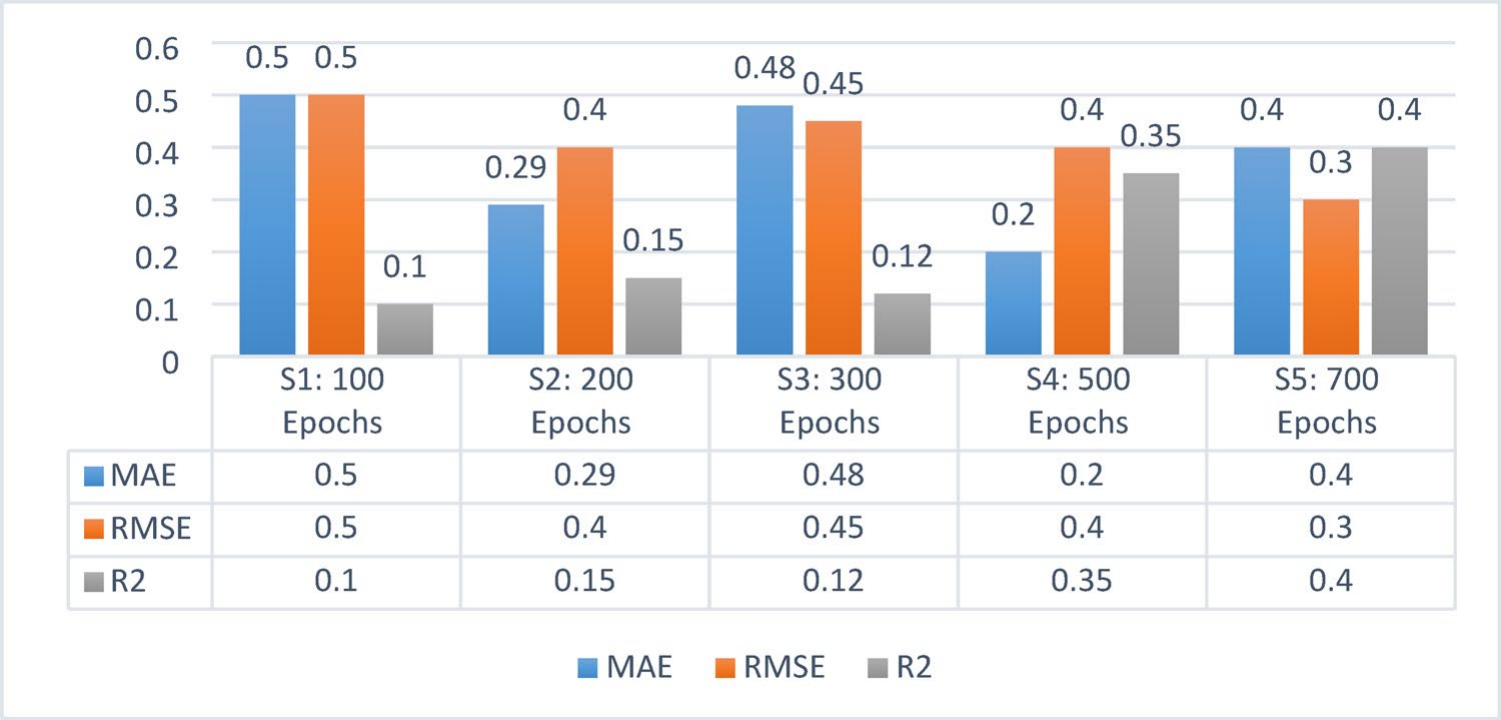
MAE, RMSE, R^2^ for GAT using 5 hidden layers and learning rate 0.01

Figure 11 depicts the results for five hidden layers with a dropout value of 0.2 and a learning rate of 0.01. Series 1 represents 100 epochs that gained the outcomes of MAE, RMSE, as well as R^2^ values of 0.5, 0.5, and 0.1, respectively. Series 2 represents 200 epochs that gained the outcomes of MAE, RMSE, and R^2^ values recorded at 0.29, 0.4, and 0.15, respectively. Series 3 represents 300 epochs that gained the scores of MAE, RMSE, alongside R^2^ values of 0.48, 0.45, and 0.12, respectively. Series 4 represents 500 epochs that gained the scores of MAE, RMSE, as well as R^2^ are 0.20, 0.4, and 0.35, respectively. Series 5 represents 700 epochs, for which the scores of MAE, RMSE, and R^2^ are 0.4, 0.3, and 0.4, respectively.

**Fig. 12:**
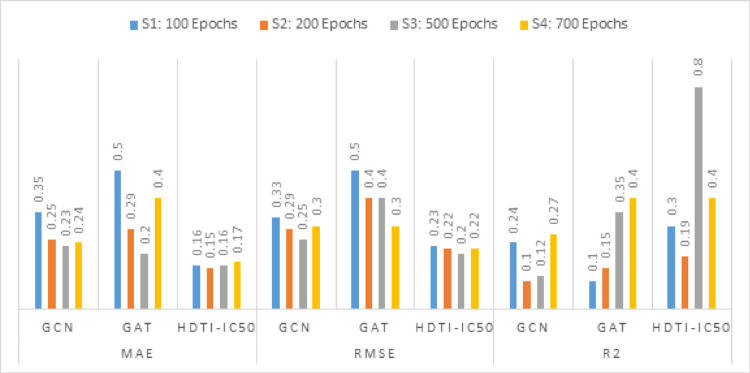
Comparative Results for HDTI-IC50, GCN, and GAT using MAE, RMSE, and R^2^ for 5 hidden layers and a learning rate of 0.01

Figure 12 depicts the results for five hidden layers configured with a dropout rate equals 0.2 and a learning rate equals 0.01. It represents the values for 100, 200, 500, and 700 Epochs that gained the scores of MAE, RMSE, as well as R^2^ for GCN and GAT.

HDTI-IC50 consistently demonstrated superior performance compared to both GCN and GAT across all training epochs. At 500 epochs, the proposed model achieved its best results, with MAE = 0.16, RMSE = 0.20, and R^2^ = 0.80, outperforming the best configurations of both GCN and GAT in all evaluation metrics. The model also maintained stable and competitive performance across other epoch settings, indicating its robustness and generalization capability.

To further assess the effectiveness of the proposed model, a comparison is conducted against five traditional regression algorithms. Table 12 presents the comparative performance of five regression models — Deep Neural Network (DNN), Support Vector Regressor (SVR), Lasso Regression, Linear Regression (LR), and K-NN Regressor — in predicting IC50 values, evaluated using the previously discussed evaluation metrics. The DNN model recorded the highest error values (MAE = 0.3, RMSE = 0.4) with a relatively low value (R^2^ = 0.70). The SVR model achieved a substantially lower error (MAE = 0.25, RMSE = 0.30) but with a low R^2^ of 0.18, showing limited variance explanation. Lasso Regression produced similar results (MAE = 0.26, RMSE = 0.32, R^2^ = 0.10). LR demonstrated a significant improvement in prediction accuracy (MAE = 0.25, RMSE = 0.20) with an R^2^ of 0.78. The K-NN Regressor performed comparably well (MAE = 0.23, RMSE = 0.45, R^2^ = 0.79). Notably, the proposed HDTI-IC50 model outperformed all baselines, achieving the lowest error values (MAE = 0.10, RMSE = 0.20) and the highest R^2^ (0.80), indicating superior predictive accuracy and generalization ability.

**Table 12:** MAE, RMSE, and R^2^ of five regression models —DNN, SVR, Lasso Regression, Linear LR, and K-NN Regressor

	MAE	RMSE	R^2^
DNN	0.3	0.4	0.7
SVR	0.25	0.3	0.18
Lasso regression	0.26	0.32	0.1
LR	0.25	0.2	0.78
K-NN regressor	0.23	0.29	0.79
HDTI-IC50	0.1	0.19	0.8

**Fig. 13:**
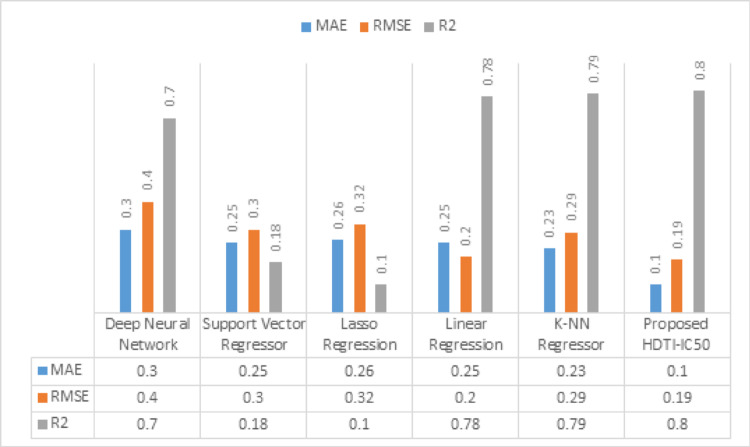
Comparative performance of the proposed HDTI-IC50 model against baseline regression models. The bar chart illustrates MAE, RMSE, and R^2^ values for each model. Lower MAE and RMSE indicate better prediction accuracy, while higher R^2^ reflects stronger correlation between predicted and actual IC50 values

Figure 13 presents a comparative analysis of the proposed HDTI-IC50 model against five baseline regression approaches: DNN, SVR, Lasso Regression, LR, and K-NN Regressor. The proposed model achieves the lowest error metrics (MAE = 0.1, RMSE = 0.19) and the highest coefficient of determination (R2 = 0.80), surpassing all other methods. While the K-NN Regressor achieved a relatively high R2 score of 0.79, its error rates remained higher (MAE = 0.23, RMSE = 0.29) compared to HDTI-IC50. The results demonstrate that the proposed architecture provides more precise and reliable IC50 predictions than both traditional ML and deep regression models.

The proposed HDTI-IC50 achieved the lowest MAE (0.10) and RMSE (0.20), outperforming all baselines. 56.5% improvement in MAE and 31.0% improvement in RMSE over the K-NN Regressor. 60.0% improvement in MAE compared to Linear Regression, with equal RMSE (0.20) but higher R2 (0.80 vs. 0.78). 66.7% improvement in MAE and 52.5% improvement in RMSE compared to the Deep Neural Network. 60.0% improvement in MAE and 33.3% improvement in RMSE over the Support Vector Regressor. 58.3% improvement in MAE and 33.3% improvement in RMSE over the Lasso Regression.

### Comparison with existing methods

The proposed **HDTI-IC50** model is compared against recent graph- and sequence-based drug–target affinity prediction models. Nguyen et al. [23] present GraphDTA, a novel deep learning model for predicting drug-target affinity (DTA) using graph neural networks. They used a graph neural network with two hidden layers at a combined GAT-GCN layers. With no. of epochs is 500, learning rate value is 0.0005, 2 number of layers and batch size is 512, table presents the comparative performance of HDTI-IC50 model and GraphDTA model. As summarized in Table 13, the proposed HDTI-IC50 achieved lower prediction errors (MAE = 0.16, RMSE = 0.19, CI = 0.67) and a substantially higher coefficient of determination (R^2^ = 0.80) compared to GraphDTA (MAE = 0.18, RMSE = 0.20, R^2^ = 0.28, CI = 0.50). For GraphDTA model the inference time is 7.51 s whereas the HDTI-IC50 model takes 7.70 s. These results indicate that HDTI-IC50 captures the underlying relationships between molecular structures and bioactivity values more effectively. The improvement in R^2^ demonstrates a better fit of the predicted IC50 values to the experimental data, reflecting stronger generalization.

**Table 13:** Comparison of HDTI-IC50 and GraphDTA models in terms of MAE, RMSE, R^2^, CI, and inference time

	MAE	RMSE	R^2^	CI	Inference time
HDTI-IC50	0.16	0.19	0.8	0.67	7.70
GraphDTA	0.18	0.22	0.28	0.50	7.51

We further evaluated the proposed HDTI-IC50 model against the recent GS-DTA framework [19]. GS-DTA predicting DTA based on graph and sequence models. According to Table 14, GS-DTA measured an RMSE of 0.20 and a concordance index (CI) of 0.63. In our studies, the HDTI-IC50 model had a slightly lower RMSE of 0.19 and a larger CI of 0.67, signifying better stability and predictability. The rise in CI reinforces the model's improved capacity to generalize beyond previously unseen pairings and highlights its applicability for enormous virtual screening tasks.

**Table 14:** Comparison of HDTI-IC50 and GS-DTA models in terms of RMSE and CI

	RMSE	CI
HDTI-IC50	0.19	0.67
GS-DTA	0.20	0.63

Furthermore, in comparison with advanced graph-based architecture such as GCN, GAT, GraphDTA and GS-DTA, the HDTI-IC50 model not only maintained higher prediction stability across different hyperparameter settings but also delivered better overall performance metrics. Reinforcing its effectiveness in capturing underlying patterns in drug–target interactions without the need for multi-modal inputs. Overall, these findings confirm that HDTI-IC50 provides a state-of-the-art solution for IC50 prediction, combining high predictive accuracy with strong generalization capabilities, making it a promising tool for drug–target interaction modeling. Table 15 summarizes the performance of all evaluated models using fivefold cross-validation and 95% confidence intervals.

**Table 15:** Comparative performance of HDTI-IC50 and baseline regression and graph-based models using fivefold cross-validation and 95% confidence intervals

	5 Folds cross validation			95% Confidence interval		
	MAE	RMSE	R^2^	MAE	RMSE	R^2^
HDTI-IC50	0.174 ± 0.005	0.251 ± 0.015	0.722 ± 0.143	0.173 ± 0.007	0.251 ± 0.020	0.701 ± 0.15
GCN	0.205 ± 0.012	0.269 ± 0.018	0.148 ± 0.061	0.205 ± 0.017	0.269 ± 0.025	0.148 ± 0.084
GAT	0.188 ± 0.008	0.258 ± 0.011	0.159 ± 0.058	0.188 ± 0.011	0.258 ± 0.016	0.159 ± 0.080
Graph-DTA	0.182 ± 0.003	0.253 ± 0.013	0.65 ± 0.023	0.182 ± 0.004	0.253 ± 0.018	0.65 ± 0.033
DNN	0.199 ± 0.044	0.215 ± 0.044	0.762 ± 0.175	0.199 ± 0.038	0.215 ± 0.038	0.662 ± 0.154
Linear regression	0.187 ± 0.012	0.199 ± 0.012	0.746 ± 0.022	0.187 ± 0.002	0.199 ± 0.010	0.746 ± 0.019
Lasso regression	0.178 ± 0.004	0.243 ± 0.015	0.65 ± 0.006	0.168 ± 0.003	0.243 ± 0.013	0.65 ± 0.005
k-NN regressor	0.184 ± 0.008	0.280 ± 0.015	0.676 ± 0.032	0.184 ± 0.001	0.288 ± 0.013	0.676 ± 0.028
Support vector regressor	0.194 ± 0.008	0.225 ± 0.013	0.193 ± 0.028	0.154 ± 0.002	0.225 ± 0.012	0.193 ± 0.024

### Interpretability of model predictions using attention mechanisms

Interpreting model predictions is crucial for understanding the decision-making process, especially in applications like IC50 value prediction. To enhance interpretability, the attention mechanism is applied to the output of the GAT layers to identify the most informative molecular substructures contributing to the prediction. Attention weights are extracted for each edge in the molecular graph by accessing the learned coefficients from the GAT layers after training. These weights were then normalized and mapped to a color scale for visualization purposes.

In practice, the attention weights can be extracted from the trained GAT model as follows:

1. Pass the molecular graph through the trained model to obtain the attention coefficients.
2. Retrieve the coefficients from the GAT layer parameters (e.g., model.gat_layer.alpha).
3. Normalize the weights to the range [0, 1].
4. Visualize the molecular graph using a library using NetworkX, coloring edges according to their attention values.

**Fig. 14:**
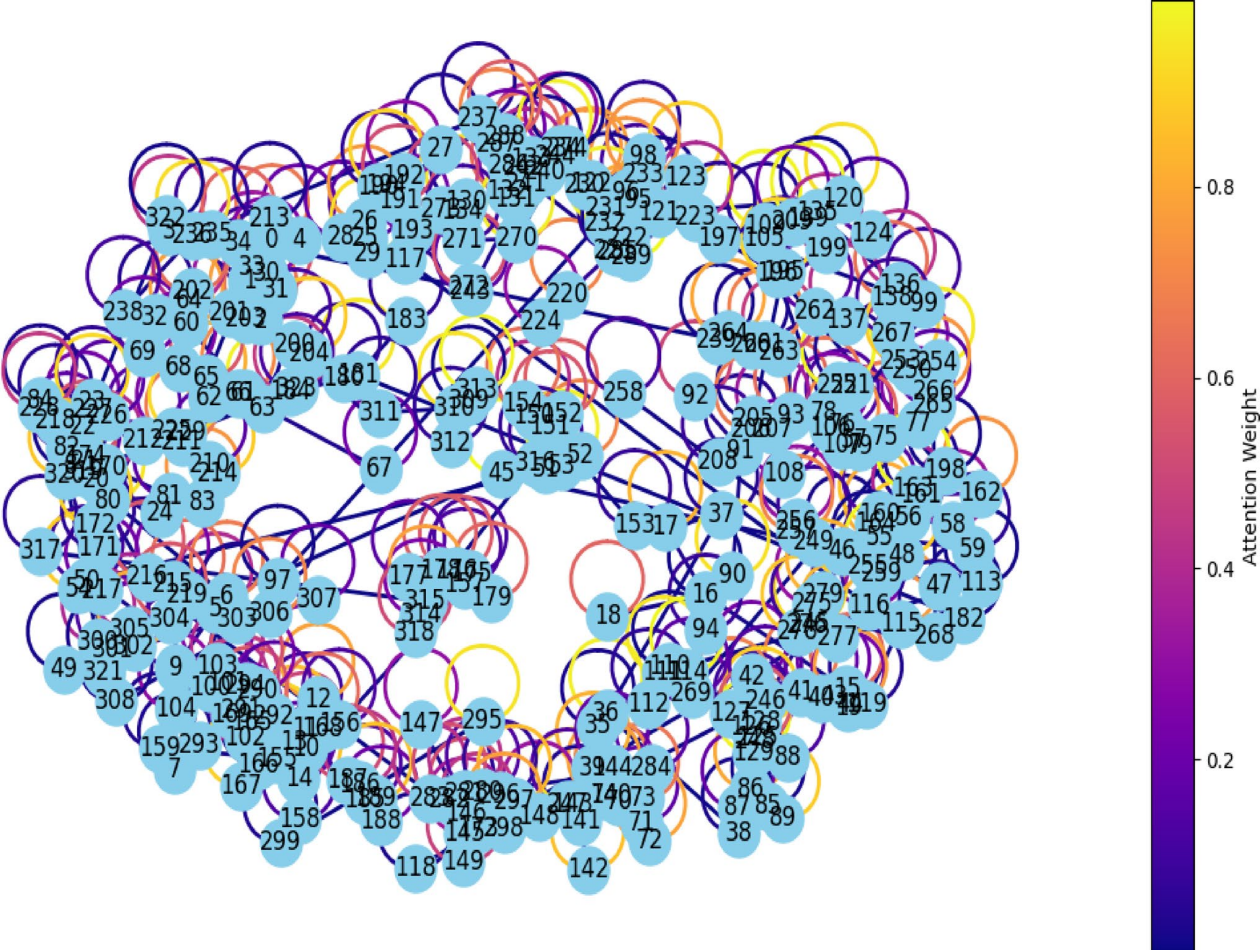
Nodes attention weight representation

Figure 14 illustrates attention weight representation, where each node corresponds to an atom, and the edges represent chemical bonds between atoms. The colors of the edges indicate the attention weights learned from the GAT. Darker blue edges correspond to lower attention weights, indicating less influence on the prediction, whereas lighter colors (yellow) represent higher attention weights, suggesting that these bonds are more informative for predicting the IC50 value. The color bar on the side maps the range of attention weights, ranging from lowest to highest. This visualization demonstrates the model’s ability to focus on specific substructures within the molecule that are most pertinent to the interaction between the medicine and the target, thereby enhancing interpretability.

## Conclusion and future work

This paper has been proposed an innovative Hybrid Drug-Target Interaction – IC50 (HDTI-IC50) prediction model, designed to investigate p53 inhibitors and predict the IC50 values. The (HDTI-IC50) model is a hybrid model that stacks GCNs and GATs to capture both local structural patterns and long-range dependencies within molecular graphs derived from SMILES strings after converting them into a graph representation. Following the GCN and GAT layers, a global pooling mechanism is applied that combines GMP and GAP to produce a fixed-size, graph-level representation. This dual aggregation strategy enables the model to capture both the most prominent and average node-level features, effectively preserving localized and holistic graph characteristics.

The HDTI-IC50 model was developed using the PyTorch framework along with the PyTorch Geometric library and trained with optimized hyperparameters. The dataset applied in this paper was sourced from the Genomics of Drug Sensitivity in Cancer database, and the model’s performance was evaluated using standard regression metrics, encompassing MAE, RMSE, and R^2^. The results indicate that HDTI-IC50 outperforms models based solely on GCN, GAT, DNN, and ML architecture, confirming its ability to learn richer node representations by stacking GCN and GAT layers. Notably, the model achieves an average inference time of 7.70 s, highlighting its computational efficiency alongside its predictive accuracy. This balance makes HDTI-IC50 well-suited for deployment in practical, resource-constrained, or latency-sensitive environments. Overall, HDTI-IC50 holds strong potential to enhance drug–target interaction predictions and support the development of novel therapeutic strategies targeting critical proteins like p53.

The HDTI-IC50 model has significant promise for real-life applications in biological drug development and discovery process. The model may be incorporated into virtual high-throughput screening pipelines to choose substances with the best effectiveness and safety by accurately anticipating IC50 values. Furthermore, it has the potential to enhance drug repurposing efforts by estimating the inhibitory potency of current drugs against new targets, thus speeding up therapeutic repositioning. Moreover, the model's predictive abilities can aid in personalized medicine, where drug efficacy may differ among individuals due to genetic and molecular differences. Also, the HDTI-IC50 framework can be combined with automated laboratory systems to facilitate AI-driven decision-making in pharmaceutical research, minimizing both the time and cost of performing experimental assays.

Even though the HDTI-IC50 model proves successful in predicting IC_50_ values, it has imperfections. Currently, the model is based on molecular graph structures and doesn’t include other biological data like gene expression patterns. The main concern lies in its reliance on general features that are readily available, such as SMILES representations and amino acid sequences. In future work, we plan to enhance the model by integrating complementary biological features to further improve prediction accuracy and biological relevance.

Future research will seek to expand the capabilities of the suggested HDTI-IC50 model in a variety of important areas. First, to improve the model's biological interpretability and prediction accuracy, the incorporation of new biological and pharmacological data sources will first be investigated. Second, transfer learning and fine-tuning methodologies will be used to improve the model's generalization performance across diverse drug-target datasets. The third step is to examine the molecular mechanisms that impact the model's predictions using explainable AI (XAI) techniques, such as attention weight visualization. Furthermore, enhancing the hybrid architecture using parallel computing and deep learning techniques as illustrated in [24] will allow for effective use of large-scale datasets and faster model training, particularly when constructing complex gene regulatory networks.

## Data Availability

The datasets analyzed during this current study are obtained from The Genomics of Drug Sensitivity in Cancer and available at the link (https://www.cancerrxgene.org/). To process the drug features, and the SMILES are transformed into molecular graphs using the freely available chemical informatics package DeepChem (https://deepchem.readthedocs.io/en/latest/api_reference/featurizers.html).

## Abbreviations

HDTI-IC50: Hybrid drug-target interaction -IC50
DTIs: Drug target interactions
IC50: Half-maximal inhibitory concentration
AI: Artificial intelligence
ML: Machine learning
FDA: Food and drug administration
DILI: Drug-induced liver injury
GDSC: GDSC (Genomics of drug sensitivity and cancer) dataset
SMILES: Simplified molecular-input line-entry
GNN: Graph neural network
GCN: Graph convolution network
GAT: Graph attention network
CNN: Convolutional neural network
GMP: Global max pooling
GAP: Global average pooling
RF: Random forest
SVM: Support vector machine
DNN: Deep neural network
LR: Linear regression
SVR: Support vector regression
PPI: Protein–Protein interaction
$\text{MAE}$: Mean absolute error
$\text{RMSE}$: Root mean square Error
${\text{R}}^{2}$: Coefficient of determination
CI: Confidence Interval

## References

[1] Annett S (2021) Pharmaceutical drug development: high drug prices and the hidden role of public funding. Biol Futura 72:129–138. 10.1007/s42977-020-00025-5 10.1007/s42977-020-00025-5 34554467

[2] Anusuya S, Kesherwani M, Priya KV, Vimala A, Shanmugam G, Velmurugan D, Gromiha MM (2018) Drug-target interactions: prediction methods and applications. Curr Protein Pept Sci 19:537–561 27829350 10.2174/1389203718666161108091609

[3] Attwood MM, Jonsson J, Rask-Andersen M, Schiöth HB (2020) Soluble ligands as drug targets. Nat Rev Drug Discov 19:695–710 32873970 10.1038/s41573-020-0078-4

[4] Bhatti UA, Tang H, Wu G, Marjan S, Hussain A (2023) Deep learning with graph convolutional networks: An overview and latest applications in computational intelligence. Int J Intell Syst 2023:1–28

[5] Blanco-González A, Cabezón A, Seco-González A, Conde-Torres D, Antelo-Riveiro P, Piñeiro Á, Garcia-Fandino R (2023) The role of AI in drug discovery: challenges, opportunities, and strategies. Pharmaceuticals 16:891. 10.3390/ph16060891 37375838 10.3390/ph16060891 PMC10302890

[6] Boutelle AM, Attardi LD (2021) p53 and tumor suppression: it takes a network. Trends Cell Biol 31:298–310 33518400 10.1016/j.tcb.2020.12.011 PMC7954925

[7] Devipriya S, Vijaya MS (2024) Graph convolutional neural network for IC50 prediction model using amyotrophic lateral sclerosis targets. In: Nanda SJ, Yadav RP, Gandomi AH, Saraswat M (eds) Data science and applications. Springer Nature, Singapore, pp 77–91. 10.1007/978-981-99-7820-5_7

[8] D’Souza S, Prema KV, Balaji S (2020) Machine learning models for drug–target interactions: current knowledge and future directions. Drug Discov Today 25:748–756 32171918 10.1016/j.drudis.2020.03.003

[9] El-Behery H, Attia A-F, El-Fishawy N, Torkey H (2021) Efficient machine learning model for predicting drug-target interactions with case study for Covid-19. Comput Biol Chem 93:107536 34271420 10.1016/j.compbiolchem.2021.107536 PMC8256690

[10] Feng L, Fu X, Du Z, Guo Y, Zhuo L, Yang Y, Cao D, Yao X (2025) MultiCTox: empowering accurate cardiotoxicity prediction through adaptive multimodal learning. J Chem Inf Model 65:3517–3528. 10.1021/acs.jcim.5c00022 40145660 10.1021/acs.jcim.5c00022

[11] Glendening ED, Weinhold F (2021) Pauling’s conceptions of hybridization and resonance in modern quantum chemistry. Molecules 26:4110 34299384 10.3390/molecules26144110 PMC8303469

[12] Guan J, Dong D, Xie P, Zhao Z, Guo Y, Lee T-Y, Yao L, Chiang Y-C (2025) StackDILI: enhancing drug-induced liver injury prediction through stacking strategy with effective molecular representations. J Chem Inf Model 65:1027–1039. 10.1021/acs.jcim.4c02079 39786982 10.1021/acs.jcim.4c02079

[13] Huang F, Pan F, Wang L, Xiao Z, He J, Yan M, Wang J, Qiu W, Liu M, Dong H (2022) The interaction between citronellol and bovine serum albumin: spectroscopic, computational and thermal imaging studies. J Mol Struct 1251:131986

[14] Jiang D, Wu Z, Hsieh C-Y, Chen G, Liao B, Wang Z, Shen C, Cao D, Wu J, Hou T (2021) Could graph neural networks learn better molecular representation for drug discovery? A comparison study of descriptor-based and graph-based models. J Cheminform 13:12. 10.1186/s13321-020-00479-8 33597034 10.1186/s13321-020-00479-8 PMC7888189

[15] Jiang X, Zhu R, Li S, Ji P (2020) Co-embedding of nodes and edges with graph neural networks. IEEE Trans Pattern Anal Mach Intell 45(6):7075–7086 10.1109/TPAMI.2020.3029762 33052851

[16] Kim S, Bae S, Piao Y, Jo K (2021) Graph Convolutional Network for Drug Response Prediction Using Gene Expression Data. Math 9:772. https://www.mdpi.com/2227-7390/9/7/772

[17] Kipf TN, Welling M (2017) Semi-supervised classification with graph convolutional networks. 10.48550/arXiv.1609.02907

[18] Lee Y, Nam S (2021) Performance comparisons of AlexNet and GoogLeNet in cell growth inhibition IC50 prediction. Int J Mol Sci 22:7721 34299341 10.3390/ijms22147721 PMC8305019

[19] Luo J, Zhu Z, Xu Z, Xiao C, Wei J, Shen J (2025) GS-DTA: integrating graph and sequence models for predicting drug-target binding affinity. BMC Genomics 26:105. 10.1186/s12864-025-11234-4 39905318 10.1186/s12864-025-11234-4 PMC11792192

[20] Monem S, Abdel-Hamid AH, Hassanien AE (2025) Drug toxicity prediction model based on enhanced graph neural network. Comput Biol Med 185:109614 39721415 10.1016/j.compbiomed.2024.109614

[21] Monem S, Hassanien AE, Abdel-Hamid AH (2024) A multi-task graph deep learning model to predict drugs combination of synergy and sensitivity scores. BMC Bioinformatics 25:327. 10.1186/s12859-024-05925-0 39390357 10.1186/s12859-024-05925-0 PMC11468365

[22] Monem S, Hassanien AE, Abdel-Hamid AH (2024) A multi-view feature representation for predicting drugs combination synergy based on ensemble and multi-task attention models. J Cheminform 16:110. 10.1186/s13321-024-00903-3 39334437 10.1186/s13321-024-00903-3 PMC11438216

[23] Nguyen T, Le H, Quinn TP, Nguyen T, Le TD, Venkatesh S (2021) GraphDTA: predicting drug–target binding affinity with graph neural networks. Bioinformatics 37:1140–1147 33119053 10.1093/bioinformatics/btaa921

[24] Noor S, Awan HH, Hashmi AS, Saeed A, Khan S, AlQahtani SA (2025) Optimizing performance of parallel computing platforms for large-scale genome data analysis. Computing 107:86. 10.1007/s00607-025-01441-y

[25] Oikarinen T, Hannah D, Kazerounian S (2021) GraphMDN: Leveraging graph structure and deep learning to solve inverse problems. In: 2021 international joint conference on neural networks (IJCNN). IEEE, pp. 1–9

[26] Park A, Joo M, Kim K, Son W-J, Lim G, Lee J, Kim JH, Lee DH, Nam S (2022) A comprehensive evaluation of regression-based drug responsiveness prediction models, using cell viability inhibitory concentrations (IC50 values). Bioinformatics 38:2810–2817 35561188 10.1093/bioinformatics/btac177

[27] Patricio RP, Videira PA, Pereira F (2022) A computer-aided drug design approach to discover tumour suppressor p53 protein activators for colorectal cancer therapy. Bioorg Med Chem 53:116530 34861473 10.1016/j.bmc.2021.116530

[28] Proietti M, Ragno A, Rosa BL, Ragno R, Capobianco R (2023) Explainable AI in drug discovery: self-interpretable graph neural network for molecular property prediction using concept whitening. Mach Learn. 10.1007/s10994-023-06369-y

[29] Rubin AE (2023) Chemical reactions. Surface/volume. Springer Nature, Cham, pp 125–145. 10.1007/978-3-031-23749-2_7

[30] Shao J, Gong Q, Yin Z, Pan W, Pandiyan S, Wang L (2022) S2DV: converting SMILES to a drug vector for predicting the activity of anti-HBV small molecules. Brief Bioinform 23:bbab593 35062019 10.1093/bib/bbab593 PMC8921627

[31] Shin J, Piao Y, Bang D, Kim S, Jo K (2022) DRPreter: interpretable anticancer drug response prediction using knowledge-guided graph neural networks and transformer. Int J Mol Sci 23:13919. 10.3390/ijms232213919 36430395 10.3390/ijms232213919 PMC9699175

[32] Song W, Xu L, Han C, Tian Z, Zou Q (2024) Drug–target interaction predictions with multi-view similarity network fusion strategy and deep interactive attention mechanism. Bioinformatics 40:btae346. 10.1093/bioinformatics/btae346 38837345 10.1093/bioinformatics/btae346 PMC11164831

[33] Tran HNT, Thomas JJ, Ahamed Hassain Malim NH (2022) DeepNC: a framework for drug-target interaction prediction with graph neural networks. PeerJ 10:e13163. 10.7717/peerj.13163 35578674 10.7717/peerj.13163 PMC9107302

[34] Vaddavalli PL, Schumacher B (2022) The p53 network: cellular and systemic DNA damage responses in cancer and aging. Trends Genet 38:598–612 35346511 10.1016/j.tig.2022.02.010

[35] Vamathevan J, Clark D, Czodrowski P, Dunham I, Ferran E, Lee G, Li B, Madabhushi A, Shah P, Spitzer M (2019) Applications of machine learning in drug discovery and development. Nat Rev Drug Discov 18:463–477 30976107 10.1038/s41573-019-0024-5 PMC6552674

[36] Veličković P, Cucurull G, Casanova A, Romero A, Liò P, Bengio Y (2018) Graph Attention Networks. 10.48550/arXiv.1710.10903

[37] Wu L, Cui P, Pei J, Zhao L, Guo X (2022) Graph Neural Networks: Foundation, Frontiers and Applications. In: Proceedings of the 28th ACM SIGKDD Conference on knowledge discovery and data mining. Presented at the KDD ’22: The 28th ACM SIGKDD conference on knowledge discovery and data mining, ACM, Washington DC USA, pp. 4840–4841. 10.1145/3534678.3542609

[38] Xu F, Lin H, He P, He L, Chen J, Lin L, Chen Y (2020) A TP53-associated gene signature for prediction of prognosis and therapeutic responses in lung squamous cell carcinoma. Oncoimmunology 9:1731943. 10.1080/2162402X.2020.1731943 32158625 10.1080/2162402X.2020.1731943 PMC7051188

[39] Xu L, Ru X, Song R (2021) Application of machine learning for drug–target interaction prediction. Front Genet 12:680117 34234813 10.3389/fgene.2021.680117 PMC8255962

[40] Zawacka-Pankau JE (2022) The role of p53 family in cancer. Cancers 14:823. 10.3390/cancers14030823 35159090 10.3390/cancers14030823 PMC8833922

[41] Zhong Z, Li C-T, Pang J (2023) Hierarchical message-passing graph neural networks. Data Min Knowl Discov 37:381–408. 10.1007/s10618-022-00890-9

